# Subsurface heatwaves in lakes

**DOI:** 10.1038/s41558-025-02314-0

**Published:** 2025-04-10

**Authors:** R. Iestyn Woolway, Miraj B. Kayastha, Yan Tong, Lian Feng, Haoran Shi, Pengfei Xue

**Affiliations:** 1 School of Ocean Sciences, Bangor University, Menai Bridge, UK; 2 Great Lakes Research Center, Michigan Technological University, Houghton, MI USA; 3 Department of Civil, Environmental and Geospatial Engineering, Michigan Technological University, Houghton, MI USA; 4 School of Environmental Science and Engineering, Southern University of Science and Technology, Shenzhen, China; 5 Environmental Science Division, Argonne National Laboratory, Lemont, IL USA

**Keywords:** Limnology, Hydrology

## Abstract

Lake heatwaves (extreme hot water events) can substantially disrupt aquatic ecosystems. Although surface heatwaves are well studied, their vertical structures within lakes remain largely unexplored. Here we analyse the characteristics of subsurface lake heatwaves (extreme hot events occurring below the surface) using a spatiotemporal modelling framework. Our findings reveal that subsurface heatwaves are frequent, often longer lasting but less intense than surface events. Deep-water heatwaves (bottom heatwaves) have increased in frequency (7.2 days decade^−1^), duration (2.1 days decade^−1^) and intensity (0.2 °C days decade^−1^) over the past 40 years. Moreover, vertically compounding heatwaves, where extreme heat occurs simultaneously at the surface and bottom, have risen by 3.3 days decade^−1^. By the end of the century, changes in heatwave patterns, particularly under high emissions, are projected to intensify. These findings highlight the need for subsurface monitoring to fully understand and predict the ecological impacts of lake heatwaves.

## Abstract (web summary)

Heatwaves in lakes are increasing with climate change, but are typically studied at the surface; little is known about heatwave dynamics with depth. This study finds subsurface heatwaves last longer, but are less intense than surface heatwaves and have increased in frequency over the past 40 years.

## Main

Lake heatwaves, prolonged periods of anomalously warm water events, have recently become distinguishable features of lake temperature variability. Studies have demonstrated that climate change has driven a notable increase in the frequency, duration and intensity of these hot extremes^1^. Model projections suggest that, during the twenty-first century, lake heatwaves are likely to intensify, become longer lasting and their occurrence frequency is expected to increase^1–3^. The rise of unprecedented temperatures during a lake heatwave can benefit some aquatic species by expanding their thermal habitat^4^, but can be detrimental for others, particularly to those that live in regions close to their thermal limit^5–7^. Quantifying changes in lake heatwaves is thus critically important to anticipate the likely impact of climatic warming on lakes.

Previous studies investigating the impacts of climate change on lake heatwaves have focused on surface conditions^1,2^. Most notably, lake heatwaves have been defined, like marine heatwaves^8–11^, as periods in which surface water temperatures increase above a seasonally varying 90th percentile threshold^1^. This one-dimensional (temporal) approach for describing a lake heatwave is important, not only for understanding how extreme lake surface conditions respond to climate change, but also for anticipating the likely impact of these extreme heat events on aquatic organisms that cannot or will not move. However, mobile aquatic species can respond to environmental disruptions, such as extreme surface temperatures, by relocating to favourable habitats^12–14^. In stratifying systems, bottom waters are often cooler than the lake surface and, if other environmental factors are favourable, many aquatic species could migrate to these deeper layers to escape surface thermal stress^15^. Moreover, the thermal response of lakes to climate change can differ considerably between surface and bottom waters, with the latter often, although not always^16,17^, experiencing a somewhat muted climatic response^18–20^. In turn, cooler water at depth could provide a potential thermal refuge for aquatic species as surface heatwaves become more common and intense^1,14^. However, unlike in marine systems^21–24^, the vertical dimension of lake heatwaves has not yet been considered, thus limiting our understanding of how lake environments below the water surface are responding to a more extreme world.

This study aims to fill this knowledge gap by investigating the depth to which thermal anomalies associated with lake surface heatwaves penetrate, as well as exploring how this has changed over the historic period (1980–2022) and will probably change in the future (2080–2099). This study also introduces (1) the concept of subsurface heatwaves, defined as periods in which depth-specific water temperatures reach extreme levels and (2) the presence of a vertical thermal escape, instances where aquatic species could theoretically move to deeper water to escape the thermal stress of lake surface heatwaves. By focussing on key metrics used to describe the severity of lake heatwaves—their average duration, average intensity and cumulative intensity—this study evaluates how these extreme events have changed. A multimodel approach is followed to investigate changes in lake surface and subsurface heatwaves. To quantify these changes, simulated water temperature profiles available from ISIMIP2b^25^ were investigated, as well as lake temperatures simulated from a suite of independently developed one-dimensional lake models. Moreover, to capture changes in some of the largest lakes of the world, and to investigate within-lake variations in heatwaves, the outputs from a three-dimensional (3D) model of the Laurentian Great Lakes of North America^26^ was investigated.

## A vertical thermal escape from lake surface heatwaves

**Fig. 1:**
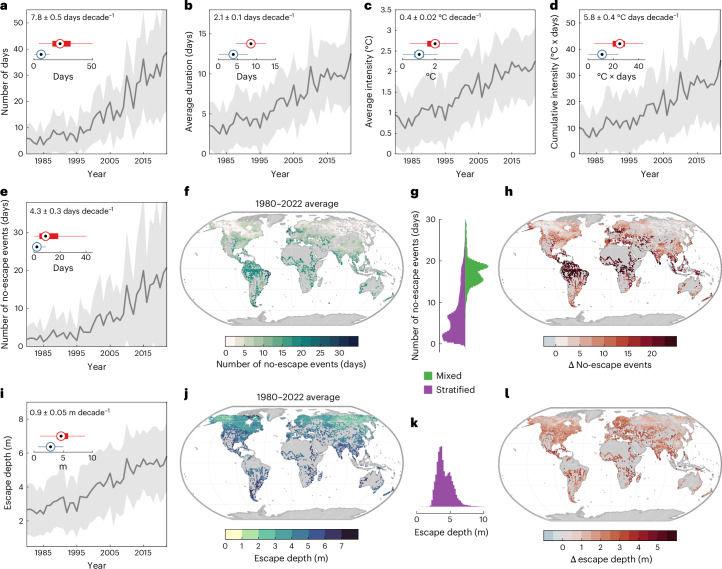
Lake surface heatwaves under climate change. **a**–**d**, Simulated changes in occurrence (**a**), duration (**b**), intensity (**c**) and cumulative intensity (**d**) of lake surface heatwaves from 1980 to 2022. **e**–**h**, Changes in no-escape events for aquatic species, including days without thermal escape (**e**), spatial variability in events (**f**), histogram comparisons between mixed and stratifying lakes (**g**) and overall change in events (2000–2022 relative to 1980–1999) (**h**). **i**–**l**, The vertical escape depth: temporal variability (**i**), spatial variability (**j**), histogram for stratified lakes (**k**) and overall change (**l**). Boxplots (*n* = 16,455) summarize changes (blue/red: first/second periods), with the central mark indicating the median and the bottom and top edges of the boxes indicating the 25th and 75th percentiles, respectively. The whiskers extend to the most extreme data points not considered outliers; shaded regions in the time series represent standard deviation and the solid line represents the mean; trends are shown in the top-left corners. Basemaps in **f**, **h**, **j** and **l** made with Natural Earth.

Over the past four decades, lake surface heatwaves have occurred widely and exhibited a marked increase (Fig. 1 and Supplementary Figs. 1–4). These extreme hot events have occurred more frequently (7.8 ± 0.5 annual days decade^−1^) and experienced an increase in their average duration (2.1 ± 0.1 days decade^−1^). They have also become stronger with an increase in their average (0.4 ± 0.02 °C decade^−1^) and cumulative (5.8 ± 0.4 °C days decade^−1^) intensity (Fig. 1a–d and Supplementary Fig. 1). These lake surface heatwave metrics have undergone even greater change in some individual lakes (Supplementary Fig. 3 and Supplementary Tables 1–3), with prominent alterations in the Laurentian Great Lakes (Supplementary Fig. 4). Lake surface heatwaves in the Great Lakes have occurred more frequently (7.5 ± 1.03 annual days decade^−1^), become longer lasting (1.4 ± 0.36 days decade^−1^) and experienced an increase in their average (0.01 ± 0.03 °C decade^−1^) and cumulative (3.7 ± 1.05 °C days decade^−1^) intensities.

**Extended Data Fig. 1:**
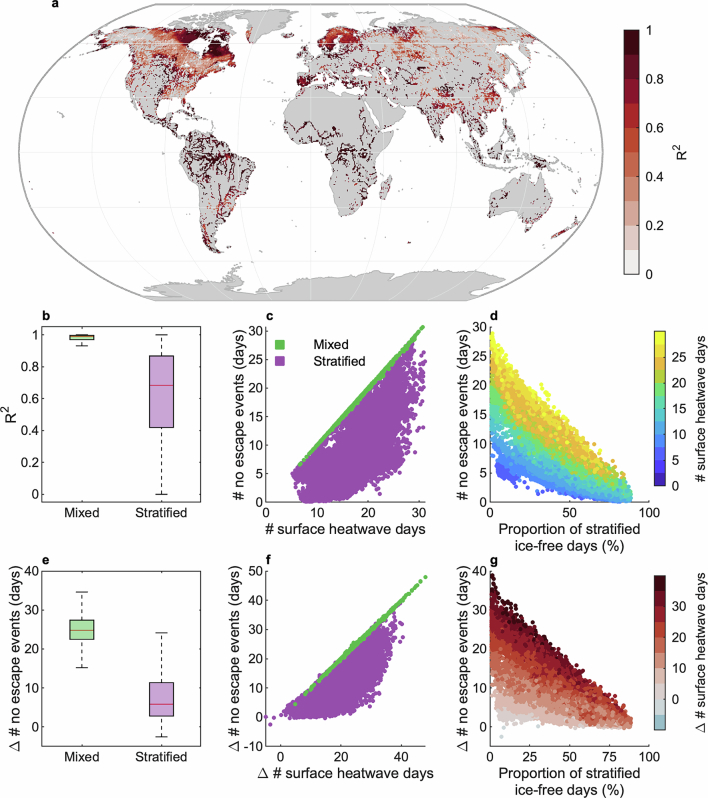
Drivers of no escape events. (**a**) Correlation between the inter-annual variability in the number of no escape events and the occurrence of surface heatwaves. (**b**) Average correlation in mixed and stratifying lakes. (**c**) Relationship between the average number of no escape events and lake surface heatwaves. (**d**) For stratifying lakes, we show the relationship between the average number of no escape events, the number of surface heatwave days, and the proportion of stratified ice-free days. (**e**) Changes in the number of no escape events between 1980-1999 and 2000-2022. (**f**) Relationship between the change in the number of no escape events and the number of surface heatwave days. (**g**) For stratifying lakes, we show the relationship between the change in the number of no escape events, the number of surface heatwave days, and the proportion of stratified ice-free days. In each box plot (n = 16,455), the central mark indicates the median, and the bottom and top edges of the boxes indicate the 25th and 75th percentiles, respectively. The whiskers extend to the most extreme data points not considered outliers. Basemap in **a** made with Natural Earth.

Our study identified the presence of a vertical thermal escape in lakes, described as instances where motile aquatic species could theoretically move to deeper water to escape surface thermal stress. Several lake surface heatwaves have occurred at times in which a vertical thermal escape could be found in deeper regions (where temperatures at depth were below the 90th percentile temperature threshold of lake surface heatwaves). From 1980 to 2022, our analysis illustrates an increase (4.3 ± 0.3 days decade^−1^) in the annual total number of lake surface heatwaves occurring without a vertical thermal escape (Fig. 1e), with even greater changes in some lakes (Supplementary Fig. 5 and Supplementary Table 4). The numbers of no-escape events in the studied lakes are greater (17.5 days) in water bodies that do not experience thermal stratification, compared to those that seasonally stratify (7.0 days) (Fig. 1f,g and Supplementary Fig. 6). This is because vertically mixed lakes lack the thermal gradient needed to separate warm surface waters from cooler, deeper layers. Consequently, during heatwaves, the entire water column warms uniformly, preventing the establishment of cooler refuges. In mixed systems, the number of no-escape events increases linearly with surface heatwave days (Extended Data Fig. 1a–d). In stratifying lakes, the relationship is variable and is related to the duration of thermal stratification (Extended Data Fig. 1d). Our analysis also suggests that changes to the number of no-escape events during the historic period (1980–1999 versus 2000–2022) have been greater in mixed (25.1 days) than in stratified (8.0 days) systems, influenced by the change in the frequency of surface heatwaves as well as a change in the proportion of stratified days (Extended Data Fig. 1e,g).

**Extended Data Fig. 2:**
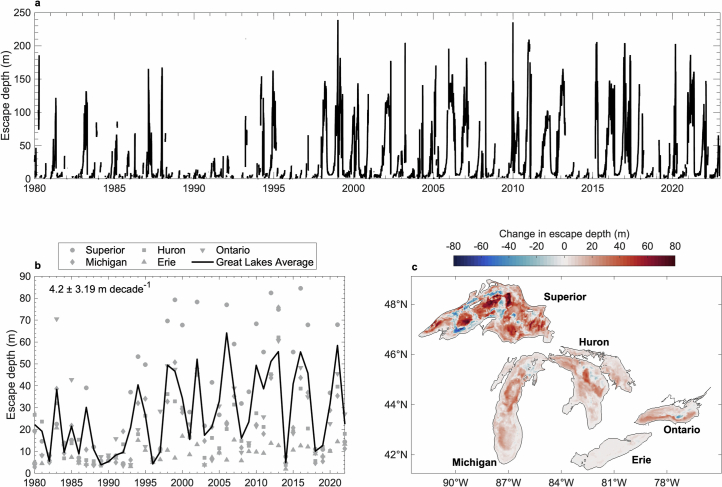
Changes in the escape depth in the Great Lakes. Shown in panels **a**-**b** are the (**a**) daily and (**b**) annual changes in escape depth in the Great Lakes. Panel **c** shows the spatial variability across the Great Lakes in terms of temporal differences (2000-2022 relative to 1980-1999) in the escape depth. Basemap in **c** made with Natural Earth.

**Extended Data Fig. 3:**
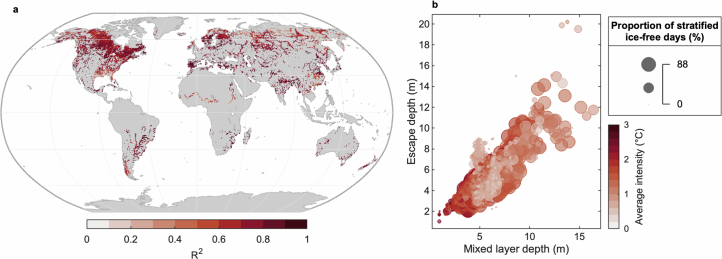
Relationship between escape depth and surface heatwave intensity. (a) Correlation (R^2^) between the inter-annual variability in the escape depth and the intensity of lake surface heatwaves. (**b**) The relationship between the average (1980-2022) escape depth, the average intensity of lake surface heatwaves, and the depth of the upper mixed layer in lakes. Basemap in **a** made with Natural Earth.

When a vertical thermal refuge exists in stratified lakes, our analysis illustrates that the vertical distance that aquatic species must travel to escape a lake surface heatwave (the escape depth) has increased (0.9 ± 0.05 m decade^−1^) (Fig. 1i). In the Laurentian Great Lakes, the escape depth has increased ~1.5-fold, from 24.7 to 38.2 m, at a rate of 4.2 ± 3.19 m decade^−1^ (Extended Data Fig. 2). However, the escape depth has remained unchanged in some lakes (Supplementary Fig. 7 and Supplementary Table 5). The interannual variability in the escape depth is positively correlated (*R*^2^ = 0.7) with the intensity of lake surface heatwaves (Extended Data Supplementary Fig. 3a and Supplementary Fig. 8a–c). On average, the escape depth is also positively related to the depth of the upper mixed layer (Methods) (Extended Data Fig. 3b and Supplementary Fig. 8d).

## Subsurface and lake bottom heatwaves

**Fig. 2:**
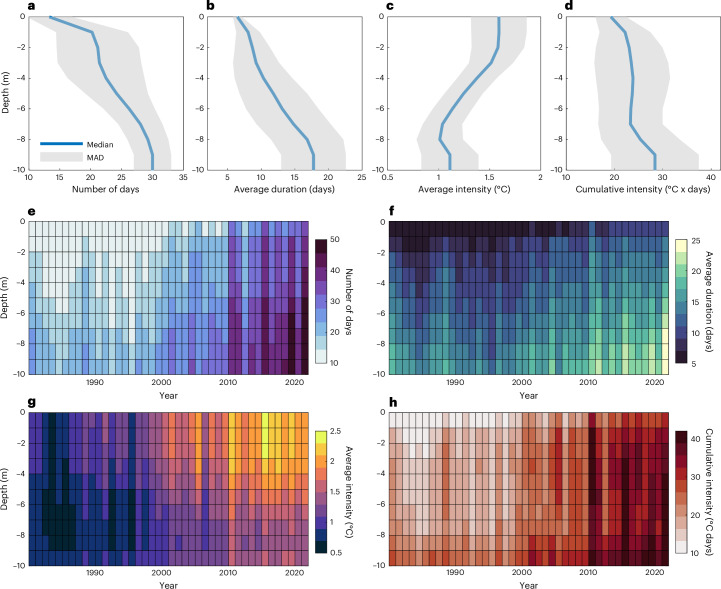
Subsurface evolution of lake heatwaves. **a**–**d**, The vertical variability of each lake heatwave metric from 1980 to 2022, with the central line representing the median and the shaded region representing the median absolute deviation (MAD). Shown as the vertical profiles of total heatwave days (**a**), average heatwave duration (**b**), average heatwave intensity (**c**) and heatwave cumulative intensity (**d**). **e**–**h**, The interannual variability in each lake heatwave metric at different depths (negative depths are below the lake surface), showing the changes with depth in total heatwave days (**e**), average heatwave duration (**f**), average heatwave intensity (**g**) and heatwave cumulative intensity (**h**). Here we compare only the vertical profiles of each heatwave metric across lakes that are shallower or equal to 10 m.

**Extended Data Fig. 4:**
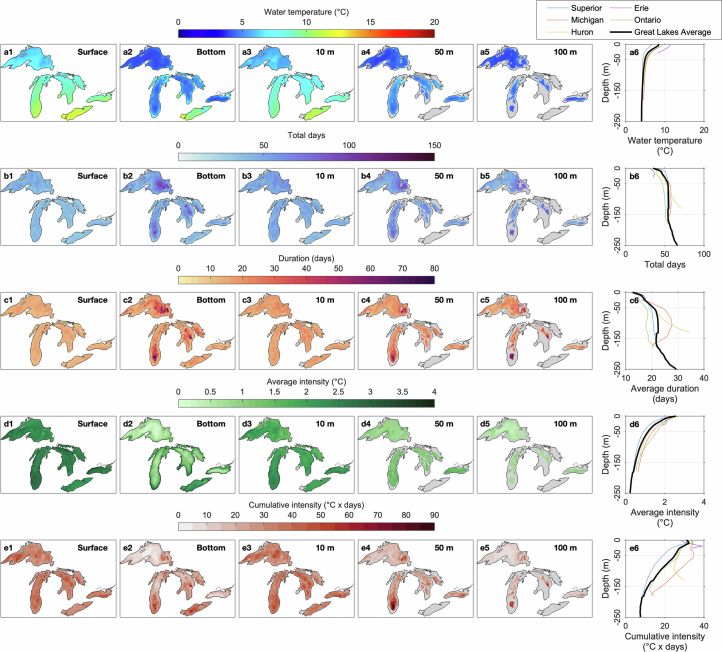
Subsurface heatwaves in the Great Lakes. Shown are the spatial variability across the Great Lakes at surface, bottom, 10 m, 50 m, and 100 m depths for **(a1-a5)** water temperature, **(b1-b5)** occurrence, **(c1-c5)** average duration, **(d1-d5)** average intensity, and **(e1-e5)** average cumulative intensity of lake heatwaves. We also show a lake-wide averaged change in **(a6)** water temperature, **(b6)** occurrence, **(c6)** average duration, **(d6)** average intensity, and **(e6)** cumulative intensity of lake heatwaves with depth. The values are the annual means during 1980-2022. Basemaps in **a**–**e** made with Natural Earth.

**Extended Data Fig. 5:**
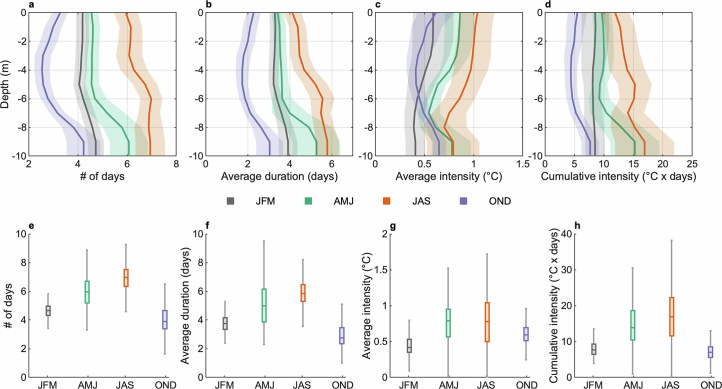
Seasonality of subsurface heatwaves. Shown in panels **a-d** are the vertical variability of each lake heatwave metric, namely their (a) occurrence, (b) average duration, (c) average intensity, and (d) cumulative intensity, from 1980 to 2022 during different seasons. The central line in each profile represents the median and the shaded region represents the median absolute deviation. In panels **e-h** we show the seasonal variability in each of the lake bottom heatwave characteristics, namely their (**e**) occurrence, (**f**) average duration, (**g**) average intensity, and (**h**) cumulative intensity during the historic period (1980-2022). In each box plot (n = 16,455), the central mark indicates the median, and the bottom and top edges of the boxes indicate the 25th and 75th percentiles, respectively. The whiskers extend to the most extreme data points not considered outliers.

**Extended Data Fig. 6:**
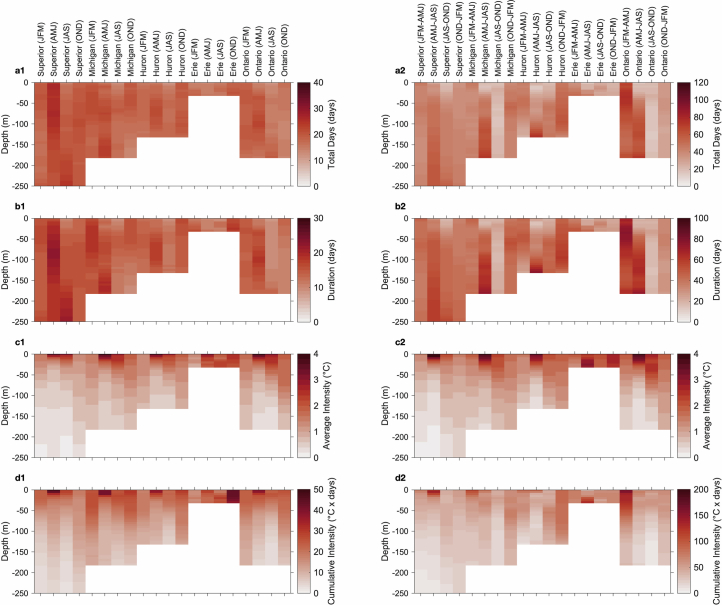
Seasonality of subsurface heatwave characteristics in the Great Lakes. Characteristics are shown for heatwave events that span a single season **(a1-d1)** and two seasons **(a2-d2)**. We show the seasonal variability in each of the lake heatwave characteristics, namely their **(a)** average occurrence, **(b)** average duration, **(c)** average intensity, and **(d)** average cumulative intensity. The values are the annual means during 1980-2022.

Subsurface heatwaves, instances of extreme hot thermal events at different depths relative to a depth-specific climatological seasonal cycle, follow a clear vertical pattern of variability in stratified lakes (Fig. 2, Supplementary Fig. 2 and Extended Data Fig. 4). The vertical structure of subsurface heatwaves can differ seasonally (Extended Data Figs. 5 and 6), typically occurring more frequently, being more intense and longer lasting during the warmer seasons (for example, April to June and July to September in the Northern Hemisphere). Annually, the total number of heatwave days is least at the surface and increases with depth (Fig. 2a, Supplementary Fig. 2a,b and Extended Data Fig. 4b). The average duration of heatwaves is also shorter at the surface and increases with depth (Fig. 2b, Supplementary Fig. 2c,d and Extended Data Fig. 4c). In contrast, our analysis suggests that the average intensity follows the opposite pattern (Fig. 2c, Supplementary Fig. 2e,f and Extended Data Fig. 4d). Regarding their cumulative intensity, which is influenced by both the duration and intensity of lake heatwaves, there are less notable relative changes with lake depth (Fig. 2d). In the Great Lakes, where the rate of change in lake heatwave intensity with depth is far greater than the change in duration, a decreasing pattern in cumulative intensity from the surface to deep waters is estimated (Extended Data Fig. 4e). These large systems also experience considerable spatial variations in subsurface heatwave characteristics (Extended Data Fig. 4). Our analysis illustrates that the vertical structure of subsurface heatwaves has changed across all stratified lakes in our study (Fig. 2e,h).

**Extended Data Fig. 7:**
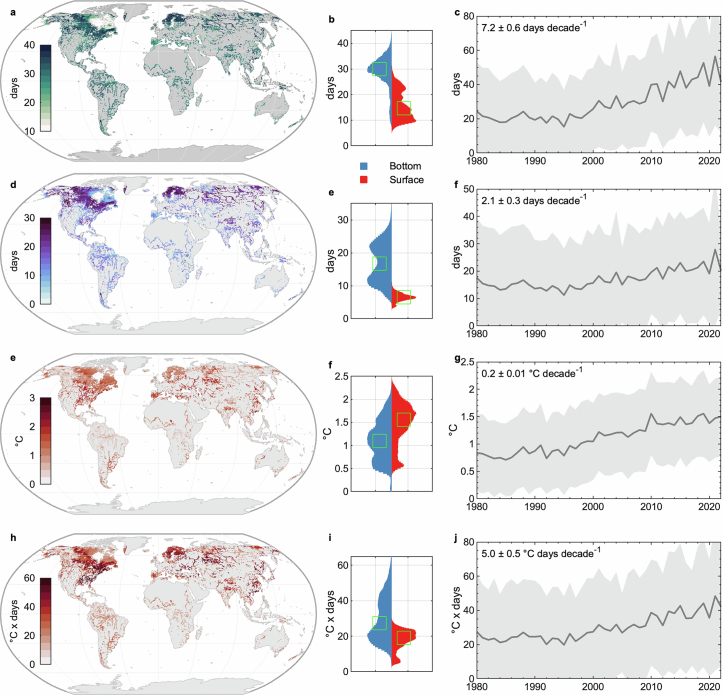
Lake bottom heatwaves under climate change. Simulated (**a-c**) occurrence, (**d-f**) duration, (**e-g**) intensity, (**h-j**) cumulative intensity of bottom heatwaves (1980-2022). Panels (**a**), (**d**), (**e**) and (**h**) show the historic average of each heatwave metric (1980-2022), with the global distribution of each bottom heatwave metric (blue) compared against those of lake surface heatwaves (red) in (**b**), (**e**), (**f**), and (**i**). In each histogram, the green square represents the median. Shown in (**c**), (**f**), (**g**) and (**j**) are the simulated changes in bottom heatwave characteristics. The solid line represents the global average, and the shaded region represents the standard deviation. Trends for each time series are shown. Basemaps in **a**,**d**,**e**,**h** made with Natural Earth.

**Extended Data Fig. 8:**
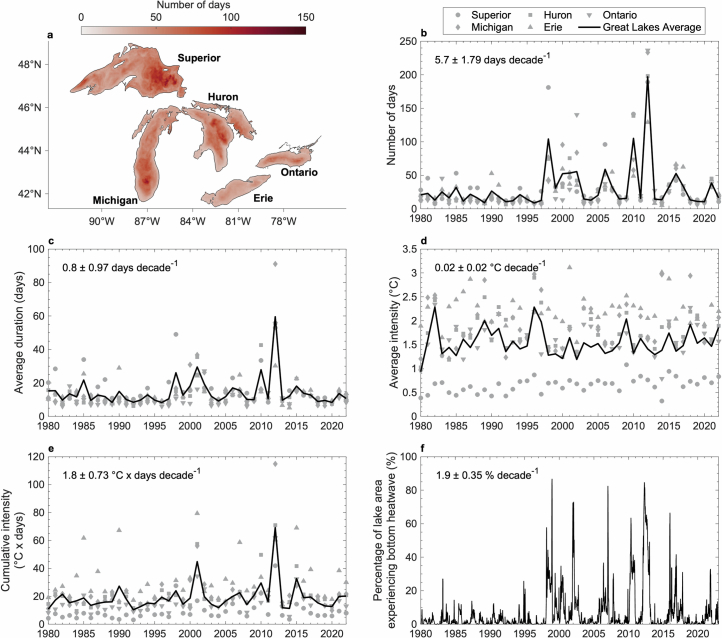
Evolution of lake bottom heatwaves in the Great Lakes. Shown are the (**a**) spatial variability across the Great Lakes for the annual mean occurrence and the simulated changes in the (**b**) occurrence, (**c**) average duration, (**d**) average intensity, and (**e**) average cumulative intensity of lake bottom heatwaves from 1980 to 2022 in the Great Lakes. Panel (**f**) shows the simulated change in the percentage of the Great Lakes area experiencing a bottom heatwave from 1980 to 2022. Basemap in **a** made with Natural Earth.

Lake bottom heatwaves are defined as extreme hot events that occur in the deepest regions of lakes. On average, the occurrence, duration and cumulative intensity of bottom heatwaves are greater than those of lake surface heatwaves (Extended Data Fig. 7). From 1980 to 2022, our analysis suggests that bottom heatwaves have increased in frequency (7.2 ± 0.6 days decade^−1^), average duration (2.1 ± 0.3 days decade^−1^), average intensity (0.2 ± 0.01 days decade^−1^) and cumulative intensity (5.0 ± 0.5 days decade^−1^). Similar changes are calculated for the Great Lakes (Extended Data Fig. 8), which have also experienced an increase (1.9 ± 0.4% decade^−1^) in the percentage coverage of lake bottom heatwaves since the 1980s. Our analysis based on individual lakes also indicates an increase in the average intensity (mean = 0.05 °C; range = −0.3–0.5 °C) of bottom heatwaves (Supplementary Fig. 3e). However, this is not always the case; in certain lakes and depths, a decreasing pattern in the occurrence days and cumulative intensity of bottom heatwaves is observed (Supplementary Fig. 3a,c,g).

**Extended Data Fig. 9:**
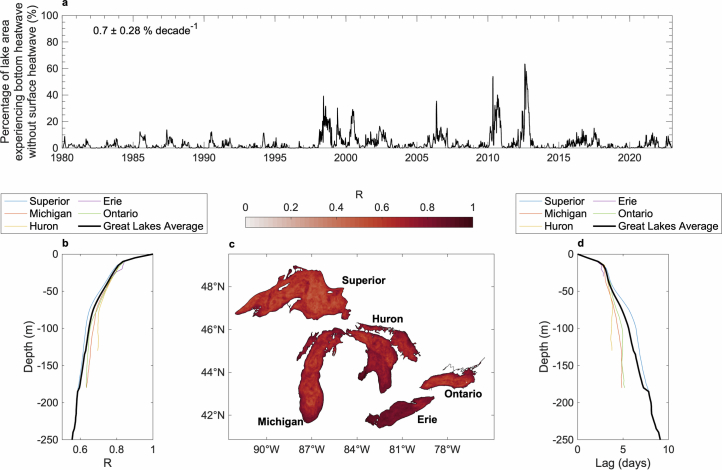
Relationship between surface and bottom heatwaves in the Great Lakes. **(a)** simulated changes in the percentage of the Great Lakes area experiencing a bottom heatwave without a surface heatwave. We also show the change in the average lagged correlation between surface and subsurface heatwave intensity with depth **(b)** and the spatial variability of the average lagged correlation between surface and subsurface heatwave intensity **(c)**. Panel **(d)** shows the statistically significant average lag identified at different depths between surface and subsurface heatwave intensities. Basemaps in **c** made with Natural Earth.

It might be assumed that bottom heatwaves occur in lakes due to the presence of a surface heatwave. However, our analysis illustrates that bottom heatwaves can occur without a lake surface heatwave (Supplementary Figs. 9 and 10 and Supplementary Table 6), the frequency of which has increased since 1980 (4.3 ± 0.3 days decade^−1^) (Supplementary Fig. 9). However, for deep lakes, it is important to note that the level of synchrony between surface and subsurface temperature anomalies varies with depth, meaning that there can be a lag (10 days) between surface and bottom heatwaves, suggesting some causality; that is, that bottom heatwaves reflect past surface heatwave conditions (Extended Data Fig. 9).

**Extended Data Fig. 10:**
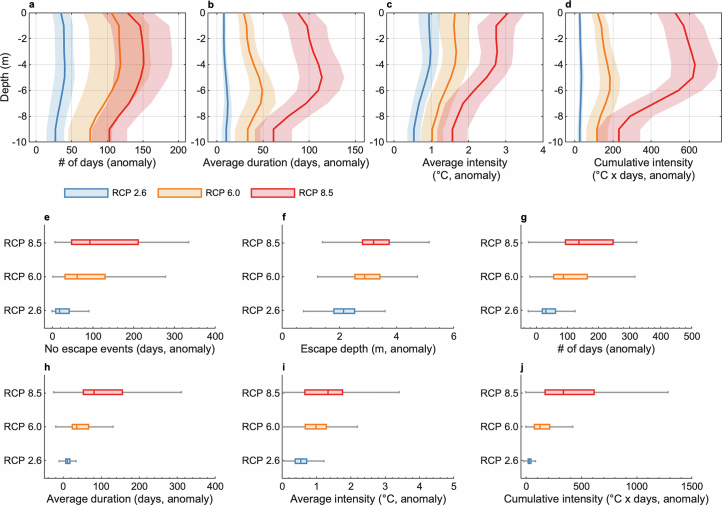
Future changes in subsurface and lake bottom heatwaves. (**a-d**) Vertical variability in lake heatwave metrics by 2080–2099 under RCPs 2.6, 6.0, and 8.5, relative to 1980-2022. Profiles include stratifying lakes that are less or equal to 10 m in depth, with medians shown as central lines and shaded regions as median absolute deviation (MAD). Panels (**e**–**j**) depict end-of-century changes in (**e**) no-escape days, (**f**) vertical escape distance, and (**g–j**) occurrence, duration, intensity, and cumulative intensity of bottom heatwaves (all lakes, including >10 m depth). In each box plot (n = 166,455), the central mark indicates the median, and the bottom and top edges of the boxes indicate the 25th and 75th percentiles, respectively. The whiskers extend to the most extreme data points not considered outliers.

Our simulations project considerable changes to subsurface and lake bottom heatwaves by the end of the twenty-first century (Extended Data Fig. 10). Future changes to lake heatwaves are prominent at the lake surface, with the magnitude of projected change then decreasing with depth. Considerable changes are also projected in the number of no-escape events, increasing by a median of between 17.3 and 92.5 days (representative concentration pathways RCP 2.6 and 8.5, respectively) and the escape depth by between 2.1 and 3.2 m (Extended Data Fig. 10e,f). In terms of lake bottom heatwaves, their occurrence (30.8–136.8 days), average duration (10.8–81.2 days), average intensity (0.5 to 1.3 °C) and cumulative intensity (27.9–334.5 °C × days) are projected to increase in the future relative to the historic period (Extended Data Fig. 10). Similarly, future heatwave projections for the Laurentian Great Lakes are consistent with global trends, showing considerable increases across all depths with large spatiotemporal variability among and within lakes, particularly under RCP 8.5 (Supplementary Figs. 11 and 12).

## Vertically compounding heatwaves

**Fig. 3:**
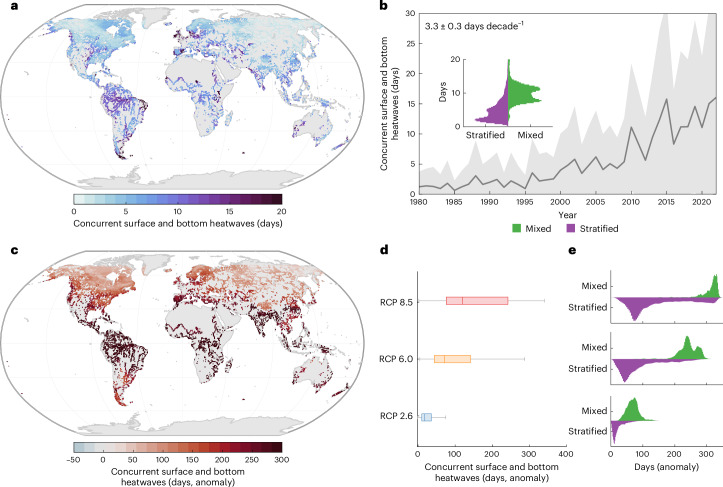
Vertically compounding heatwaves. **a**, Simulated co-occurrence of lake surface and bottom heatwaves during 1980–2022. **b**, Temporal changes in concurrent heatwaves, with global summaries for mixed and stratified lakes (inset). **c**, Projected change in co-occurrence frequency by 2080–2099 under RCP 8.5, relative to 1980–2022. **d**, Global summaries for RCP 2.6, 6.0 and 8.5. **e**, Comparison of future changes between mixed and stratified lakes. Solid lines in time series represent global averages; shaded regions show standard deviation. Boxplots (*n* = 16,455) summarize variability, with the central mark indicating the median and the bottom and top edges of the boxes indicating the 25th and 75th percentiles, respectively. The whiskers extend to the most extreme data points not considered outliers, with trends shown in **b** upper left. Basemaps in **a** and **c** made with Natural Earth.

Instances where lake heatwaves occur at either the water surface or at depth can influence ecosystem functioning. However, it could be argued that instances where these events co-occur, which we define as vertically compounding heatwaves, could have a greater impact. We estimate that vertically compounding heatwaves, which are most common during the coldest times of the year when many seasonally stratifying lakes mix (Supplementary Fig. 13) and most frequently occur in mixed systems (Fig. 3a), have increased in frequency (3.3 ± 0.3 days decade^−1^) since 1980 (Fig. 3b). Similarly, in the Great Lakes, the percentage of lake area experiencing these vertically compounding extremes has also increased (1.2 ± 0.13% decade^−1^) (Supplementary Fig. 14). Furthermore, among other individual lakes studied, 47 out of 48 lakes experienced a positive change in vertically compounding heatwaves (Supplementary Table 7). By the end of this century, vertically compounding heatwaves will become increasingly common in lakes, increasing by a median of between 16.8 and 119.1 days (RCP 2.6 and 8.5, respectively), relative to historic conditions. Vertically compounding heatwaves will become increasingly frequent in lakes that remain vertically mixed (Fig. 3c–e).

## Discussion

Lake ecosystems are undergoing transformative changes within a warming world. Our investigation further explores the thermal response of lakes to climate change, focussing on the dynamics of heatwaves across a depth gradient. Our investigation identified several findings relating to (1) the decreasing potential of a vertical thermal escape from surface heatwaves, (2) an increase in the vertical distance that species should travel to escape a surface heatwave when a thermal refuge exists, (3) considerable variability in the vertical structure of subsurface heatwaves across lakes, (4) the increased occurrence of bottom heatwaves with and without extreme surface conditions and (5) an increase in the frequency of vertically compounding heatwaves.

Previous studies have reported that lake surface heatwaves have increased in intensity and duration in recent decades^1–3^. The extreme water temperatures that species must endure during a surface heatwave can often lead to severe consequences^5,7^. However, previous studies have also described that motile aquatic species can respond to environmental disruptions, such as increasing temperatures, by relocating to favourable habitats^14^. Studies have suggested that many aquatic species will need to migrate to cooler water at higher elevation or latitude this century to maintain a preferred thermal habitat^13,27^. However, aquatic species could also escape the thermal stress of surface heatwaves by migrating to deeper regions within a lake. Our investigation demonstrates that, while there is often the potential for aquatic species to travel vertically within a lake to reach cooler water, the proportion of lake surface heatwaves without a thermal refuge in deeper water has increased. This vertical expansion of lake surface heatwaves highlights the dynamic nature of these extreme heat events, prompting aquatic organisms to adjust their distribution patterns. As the effects of lake surface heatwaves reach increasingly deeper water, a reduction in sufficient habitat can result in changes to species abundance and range.

Our understanding of lake heatwaves is dominated by extreme conditions at the lake surface. However, our study demonstrates that the subsurface layers of lakes can often exhibit heatwaves that are longer lasting and more severe. The extended duration and higher cumulative intensity of heatwaves in deeper water presents a nuanced aspect of lake thermal dynamics. Moreover, a greater intensity of bottom heatwaves implies prolonged exposure to elevated temperatures in deeper waters, potentially posing challenges for demersal organisms or bottom-dwelling fish inhabiting these regions. Another important consideration, which was not investigated in this study, is the potential for lake bottom heatwaves to influence the temperature of lake sediments, with knock-on impacts on aspects such as GHG production^17^. Studies in the marine environment have demonstrated that sediment heatwaves often co-occur with pelagic heatwaves^28^. This suggests that extremes in the overlying water column can propagate into sediments. Future research should explore this connection in freshwater systems to better understand the full ecological consequences of lake heatwaves, including the effects on sediment-dwelling species, nutrient cycling and biogeochemical processes.

Our analysis illustrated that subsurface heatwaves could occur independently of surface heatwaves as a result of the thermal structure of lakes, which can lead to the accumulation of heat in deeper layers, even in the absence of extreme surface warming. Subsurface heatwaves can arise without a surface heatwave because of time lags between surface and subsurface temperature responses. In the Great Lakes, this delay is partly caused by a pronounced difference in temperature seasonality at varying depths. While surface temperatures in the Great Lakes peak during the summer, deeper waters experience their maximum temperatures in the autumn. It is also important to note that surface heatwaves could strengthen the thermal stability of lakes and subsequently lead to a shoaling of the upper mixed layer. This increased stability could limit vertical mixing which can, in turn, restrict the transfer of heat to deeper waters and potentially hinder the development of subsurface heatwaves. However, our findings suggest that, while surface heatwaves could stabilize the thermocline and initially restrict heat transfer, prolonged periods of elevated surface temperatures could lead to the penetration of heat into deeper layers, facilitating subsurface heatwaves. To fully understand the implications of surface heatwaves on thermocline stability and the subsequent development of subsurface heatwaves, further research is needed.

Our study highlights that it is critical for resource managers to consider shifts in lake temperature extremes, particularly below the surface. We encourage that our analysis be expanded upon for individual or groups of species by incorporating additional considerations, including horizontal movements, physiology, additional essential habitat properties and other restrictions on species distributions such as the need to be near shore or specific breeding or nursing grounds. When assessing the ecological impacts of subsurface or vertically compounding heatwaves, the significance of these events will vary depending on the specific biological context. For organisms that occupy the entire water column, simultaneous surface and bottom heatwaves are particularly critical, as they can alter the thermal conditions across the entire habitat. For species that primarily inhabit surface waters, the absence of deep-water thermal refuges during surface heatwaves is of concern, as these events create extreme conditions with no possibility for escape to cooler depths. It is also important to note that, while some studies in the oceans have shown that marine heatwaves can have a detrimental impact on the ecosystem^23^, others have not shown a dominant effect^29^. Thus, when anticipating the response of aquatic species to subsurface lake heatwaves, it is important to consider that not all species will respond in the same way and that single-species responses^5^ do not suggest a net ecological effect.

The vertical expansion of heatwaves and the disparities in occurrence, duration and cumulative intensity provide valuable insights into how climate change manifests in the thermal profiles of lakes. These findings contribute to a more comprehensive understanding of the multidimensional impacts of climate change. In terms of management and conservation, our investigation advocates for an integrated approach that considers the diverse responses of surface and subsurface ecosystems to changes in extreme temperature. Protecting and restoring thermal refuges in the lower layers of lakes may be crucial for preserving biodiversity and ensuring the long-term sustainability of these ecosystems. Our analysis offers a new perspective on the vertical imprint of lake heatwaves, expanding our understanding of these extreme events and their potential impacts.

## Methods

### Large-scale simulations of lake water temperature

To simulate the occurrence of surface and subsurface lake heatwaves, we analysed daily lake temperatures provided by the ISIMIP2b lake sector. The simulations used in this study include water temperature simulations for 16,455 representative lakes worldwide, specifically those situated between 60° S and <71° N. An in-depth explanation of the ISIMIP lake sector is given by ref. ^25^. The ISIMIP2b lake sector simulations have been used previously to investigate the impact of climate change on lake heat budgets^30^, the timing and duration of summer stratification^31^ and for attributing the anthropogenic influence on lake ice and surface water temperature variations^32^. All ISIMIP lake sector projections investigated in this study were simulated via a lake model (see below) driven by an ensemble of bias-corrected climate projections—GFDL-ESM2M, HadGEM2-ES, IPSL-CM5A-LR and MIROC5—for historic and future periods, the latter under different RCPs. The data used to drive the lake models in ISIMIP2b included projections of air temperature at 2 m, wind speed at 10 m, surface solar and thermal radiation and specific humidity, which were available at a daily resolution. In this study, to obtain historic to contemporary (1980–2022) lake temperature projections, we combine the historical simulations (1980–2005) with the ‘future’ projections (2006–2022) under RCP 8.5 (refs. ^11,33,34^). Future (2080–2099) lake temperature projections were investigated under RCP 2.6 (low-emission scenario), 6.0 (medium-high) and 8.5 (high). These pathways encompass a range of potential future global radiative forcing from anthropogenic GHGs and aerosols, and results span a range of potential impacts on lake temperature.

The 16,455 representative lakes investigated in this study were simulated at a 0.5° × 0.5° grid resolution (the spatial resolution of the climate projections) with the SimStrat-UoG model. The dataset used to describe the size distribution of all lakes within each 0.5° grid has a horizontal resolution of 30 arcsec (refs. ^35,36^) and includes all known lakes equal or greater than this size threshold. Given the one-dimensional nature of the SimStrat-UoG model, we considered only lakes with a depth of <60 m, removing any deeper representative lakes from the ISIMIP2b global lake sector from the analysis. Furthermore, we included only lakes that experienced at least 2 months of ice-free conditions each year. In this study, we standardized the vertical resolution of the water temperature profiles generated by SimStrat-UoG through linear interpolation^37^. For the depth range from the surface down to 20 m, we applied a resolution of 0.1 m to capture fine-scale variations. Between depths of 20 and 50 m, the resolution was set to 0.5 m to balance detail with computational efficiency. For depths >50 m, we used a coarser resolution of 1 m to reflect the generally more uniform temperature patterns in this deeper range. This approach ensures a consistent and accurate representation of the temperature profile across different lakes.

For these representative lakes, we compare only vertical profiles of each lake heatwave metric (see below) across sites that are shallower or equal to 10 m in mean depth. These relatively shallow systems contribute to >95% of the number of studied sites, which is consistent with previous studies that have demonstrated that shallow lakes dominate the global distribution of lake types. For example, the HydroLakes dataset of ref. ^38^ suggests that ~98% of the 1.42 million lakes worldwide are ≤10 m in mean depth. While our study encompasses a broad range of lakes with varying depths, it is important to note that ~77% of the lakes have a depth of 10 m. This depth distribution implies that most of our findings, particularly those concerning deeper layers, are primarily representative of lakes with a maximum depth of ~10 m.

### Selection of individual lakes

To evaluate subsurface heatwaves in individual lakes—providing a more localized perspective than the broader ISIMIP global simulations described above—we undertook a comprehensive analysis involving 53 lakes, each with finer-scale vertical temperature profiles. This approach allows for a granular understanding of thermal dynamics at a scale that reflects individual lake characteristics rather than regional averages. Among these lakes, we included the five Laurentian Great Lakes. Owing to their vast surface area and substantial depth, temperatures in the Great Lakes were simulated using a sophisticated 3D model, which captures the complex interactions between various lake layers and provides a detailed depiction of thermal stratification and mixing processes. This high-resolution modelling is crucial for understanding the unique thermal responses of these large, deep lakes. In addition, we examined 42 lakes predominantly located in Europe and North America, for which temperature profiles were simulated using three distinct one-dimensional lake models. These models, while less complex than the 3D approach, are still capable of capturing essential vertical temperature variations and heatwave dynamics. The choice of these models was influenced by the availability of historical data and the specific thermal characteristics of these lakes. To further broaden the geographic scope and improve the spatial representation of our dataset, we included six additional lakes, some of which are situated on the Tibetan Plateau. These lakes are characterized by unique climatic and hydrological conditions, which may considerably influence their thermal behaviour. For these lakes, temperature profiles were simulated using lake-specific FLake models. The FLake model is well suited for representing thermal profiles in diverse environments, including high-altitude and remote regions, thereby enhancing the overall robustness of our analysis. By incorporating these varied modelling approaches and expanding our dataset to include lakes from different geographic and environmental settings, we aim to provide a more comprehensive understanding of subsurface heatwaves across a range of lake types and locations. This multifaceted approach ensures that our findings are not only reflective of individual lake characteristics but also contribute to a broader understanding of thermal dynamics in freshwater lakes worldwide. Each of these additional modelling approaches is described in detail within the following sections.

### Three-dimensional model-simulated lake temperature profiles for the Great Lakes

The water temperatures of the Great Lakes were simulated using the second version of the Great Lakes–Atmosphere Regional Model (GLARM)^26^, which is a two-way 3D lake ice–atmosphere coupled climate modelling system designed for the Great Lakes region. GLARM is built upon a two-way coupling between the fourth version of the International Centre for Theoretical Physics regional climate model (RegCM4) which simulates land and atmospheric processes^39^ and the finite volume community ocean model (FVCOM) which simulates the 3D lake dynamics, thermal dynamics and ice dynamics of the Great Lakes^40–42^. In the two-way coupled framework of GLARM, the Great Lakes surface temperature and ice coverage are dynamically calculated by FVCOM and are provided to RegCM4 as the lower boundary condition for the atmosphere over the Great Lakes. In turn, RegCM4 calculates and provides the surface meteorological forcing fields required by FVCOM. This two-way coupling between RegCM4 and FVCOM is achieved through the OASIS3-MCT coupler^43^, and lake hydrodynamic conditions in FVCOM are configured to evolve and freely interact with atmospheric conditions over the entire simulation.

GLARM was run for our historical study period (1980–2022) by prescribing the lateral atmospheric boundary conditions for GLARM through a combination of ERA-Interim^44^ and ERA5 (ref. ^45^) climate reanalysis data from the European Centre for Medium-Range Weather Forecasts. The lateral atmospheric boundary conditions included the 6-hourly pressure and wind components, air temperature and mixing ratio at all vertical model levels. Future (2080–2099) lake temperature projections were investigated under RCP 8.5 (high emission) with the lateral atmospheric boundary conditions provided by three general circulation models (GCMs)—IPSL-CM5A-MR, MPI-ECM-MR and GISS-E2-H. The future projections presented in this paper are the ensemble average of the three downscaled projections. The three GCMs were selected out of 19 GCMs on the basis of their performance in reproducing the observed climate and projecting future warming trends over North America^26^. GLARM modelling offers climate change projections that incorporate both the Great Lakes basin and the changes within the five Great Lakes, by integrating a two-way interaction between a regional climate model and a 3D lake model. The configuration and performance of the model, in replicating historical lake conditions, selecting GCM for dynamical downscaling and projecting future climates, are comprehensively detailed in ref. ^46^ and ref. ^26^. The comprehensive comparisons of GLARM-simulated lake temperature against mooring observation^47^ temperatures and satellite-derived temperatures are presented in Supplementary Fig. 15. The Great Lakes simulations are available from ref. ^48^.

### Ensemble model-simulated lake temperature profiles for 42 individual lakes

The ISIMIP2b lake sector provides lake-specific simulations from 42 lakes with detailed bathymetry and validation data. Lake temperatures for these lakes were simulated by a suite of independently developed lake models: (1) general lake model, (2) general ocean turbulence model and (3) SimStrat. We conducted an analysis of these simulations with the objective of studying individual lakes at a fine scale, ensuring a diverse representation across climates and lake types. For all sets of simulations, lake heatwave metrics were calculated independently for each lake–climate model combination and then averaged across the lake–climate model ensemble.

### FLake-simulated lake temperature profiles for six individual lakes

Focusing on the additional six lakes, we adopted the FLake model to simulate characteristics of lake vertical heatwaves. FLake is a heat transfer model that parameterizes the vertical temperature profiles of two-layer water, including a vertically uniform upper layer and a stably stratified lower layer consistent with self-similarity theory^49–51^. When the lake surface is covered by ice and snow, the model accounts for the presence of these layers. Owing to the balance between computational efficiency and simulation performance, FLake has been widely used for accurately reproducing surface temperatures^52^ and lake mixing regimes^53,54^ at regional and global scales.

The climate forcing variables used to drive the FLake model were obtained from the hourly grid ERA5-Land product (1981–2020), with data extracted from grid cells located at the designated lake centres. Detailed initialization instructions for lake characteristics and other model parameters required by FLake are outlined in our previous work^52^. In this study, to ensure the accurate simulation of vertical thermal conditions, we first compiled vertical temperature observations from six lakes not included in the 42 lakes provided in ISIMIP2b local simulations. These in situ observations were then leveraged to determine the optimal model settings within FLake for individual lakes. The parameters subjected to calibration in this study include snow accumulation rate (kg (m^−2^ s^−1^); ranging from 0.0000001 to 0.5), scale and offset of lake depth (m; ranging from 0.75 to 1.25 for scale and 0–20 for offset), scale and offset of wind speed (U, m s^−1^; ranging from 0.75 to 1.25 for range and 0 to 8 for offset), albedo for snow and white ice (ranging from 0.6 to 0.8), albedo for melting snow and blue ice (ranging from 0.1 to 0.6), light attenuation coefficient (Kd, m^−1^; ranging from 0.1 to 3), scale of solar radiation (ranging from 0.7 to 1.3) and scale of surface air temperature (ranging from 0.9 to 1.1).

Note that the calibration objective aimed to satisfy several error criteria, with all errors <2 °C and the minimum average of several error criteria serving as the selection criterion of optimal parameter settings. The error criteria selected for evaluation include simulation errors at various depths (full depth, surface layer, one-third depth, two-thirds depth and bottom) and across seasons (spring, summer, autumn and winter). The error metric used is the median absolute error (MAE) of daily mean temperature simulations. The comprehensive evaluation results of simulation performance for the six lakes are presented in Supplementary Table 8 and Supplementary Fig. 16. These simulations are available from ref. ^53^.

### Lake heatwave metrics

Using the daily simulated lake water temperatures, the occurrence, average duration, average intensity and cumulative intensity of lake heatwaves were estimated following the methods described by ref. ^1^. Specifically, using the R package heatwave^55^, lake heatwaves were defined as when daily lake temperatures were above a local and seasonally varying 90th percentile threshold for at least five consecutive days. Climatological mean of lake temperature was calculated for each calendar day using the daily temperatures within an 11-day window centred on the date across all years within a climatological period, here defined as 1991–2020 and smoothed by applying a 31-day moving average. In addition, two events with a break of <2 days were considered as a single event. The average duration of lake heatwaves is defined as the difference between the start and end dates of a specific heatwave; the average intensity is the average temperature anomaly relative to seasonal climatology averaged during an event; and the cumulative intensity is calculated as the sum of the temperature anomaly during the total duration of each lake heatwave. Lake heatwaves were defined exclusively during the ice-free season. Our primary focus was on examining changes in heatwave metrics on an annual (ice-free) scale, acknowledging that ecologically critical events occur throughout the year in lakes and are not limited to the warmer (for example, summer) season. However, lake heatwave characteristics were also compared across different seasons: January–March, April–June, July–September and October–December. For each seasonal comparison, only lakes that experienced a minimum of two ice-free months within the respective season were included. Subsurface heatwaves were calculated relative to a subsurface lake temperature climatology, with this climatology calculated from the temperature time-series data at specific depths. For example, a bottom lake heatwave was calculated relative to the bottom water temperature climatology.

The thermal escape depth was estimated as the depth at which water temperatures decrease below the 90th percentile threshold of a lake surface heatwave. The escape depth is crucial for anticipating how surface heatwaves might affect lake ecosystems. Surface heatwaves, which create extreme temperatures in the upper layers of a lake, can prompt aquatic species to seek cooler refuges at depth. The thermal escape depth, therefore, represents the boundary below which temperatures remain conducive to aquatic life, offering a vital refuge during periods of intense surface heating. The occurrence of vertically compounding heatwaves, defined as times in which a lake surface and bottom heatwave occurred at the same time, was also calculated. The global heatwave metrics calculated from the ISIMIP projections are available from ref. ^56^.

### Lake stratification and mixing

Understanding lake stratification is essential for analysing vertical thermal dynamics and the occurrence of subsurface heatwaves in lakes. In this study, a lake is defined as stratified if there is a temperature difference of >1 °C between the surface and the bottom water^57–59^. Lakes that do not meet this criterion are classified as mixed. In mixed lakes, where the water column is relatively homogeneous and temperature gradients between surface and bottom layers are minimal, surface heatwaves typically reflect the thermal conditions throughout the entire lake. Consequently, extreme temperature variations are generally uniform across the water column. In contrast, stratified lakes usually exhibit distinct thermal layers during the summer, including the epilimnion, thermocline and hypolimnion. In these lakes, the thermal characteristics of the surface and bottom layers can be markedly different. Although the thermocline reduces the likelihood of extreme surface temperatures directly affecting the hypolimnion, long-term trends and prolonged surface heatwaves can eventually contribute to increased thermal stress in deeper layers over time. This study compares subsurface heatwaves between stratified and mixed lakes to understand these dynamics better. Additionally, in this study the depth of the upper mixed layer was calculated by using Lake Analyzer^60^. For the Great Lakes, which are very deep dimictic lakes, the mixed layer depth was calculated as the shallowest depth where the density exceeded the surface density by 0.1 kg m^−3^ (ref. ^61^).

### Analysis

To explore potential temporal synchrony between the warming of the surface and subsurface layers, Pearson’s correlation coefficients (*R*) and lag between surface and subsurface intensities were estimated on an event-by-event basis with *P* values as a measure of statistical significance (*P* < 0.05). Correlations were calculated if a surface event overlaps with a subsurface event within a 5-day margin at the start or end of each event.

### Reporting summary

Further information on research design is available in the Nature Portfolio Reporting Summary linked to this article.

## Online content

Any methods, additional references, Nature Portfolio reporting summaries, source data, extended data, supplementary information, acknowledgements, peer review information; details of author contributions and competing interests; and statements of data and code availability are available at 10.1038/s41558-025-02314-0.

## Supplementary information

Supplementary Information Supplementary Figs. 1–16 and Tables 1–8.

Reporting Summary

## Data Availability

ISIMIP local lake simulations are available from 10.48364/ISIMIP.563533. ISIMIP global lake simulations are found at 10.48364/ISIMIP.931371. Simulations of lake temperature profiles over individual lakes generated in this study are available at ref. ^53^. The mooring observations for the Laurentian Great Lakes used in the model evaluation are available from https://www.glerl.noaa.gov/data/#watertemp. The heatwave metrics calculated for the Great Lakes are available at ref. ^48^. Global heatwave metrics calculated from the ISIMIP projections are available at ref. ^56^.

## Code availability

Code used to reproduce the analysis and figures generated for this paper are available at ref. ^56^. The source code for the FLake model is accessible at http://www.flake.igb-berlin.de/.
