# Supplementary material for: Subsurface heatwaves in lakes
Source: Nat Clim Chang. 2025 Apr 10;15(5):554–9. doi: 10.1038/s41558-025-02314-0 (PMC12064439; doi:10.1038/s41558-025-02314-0)
Supplement: Supplementary file 1 — Supplementary Figs. 1–16 and Tables 1–8. [file 41558_2025_2314_MOESM1_ESM.pdf]

nature climate change

Supplementary information https://doi.org/10.1038/s41558-025-02314-0

Subsurface heatwaves in lakes

In the format provided by the authors and unedited

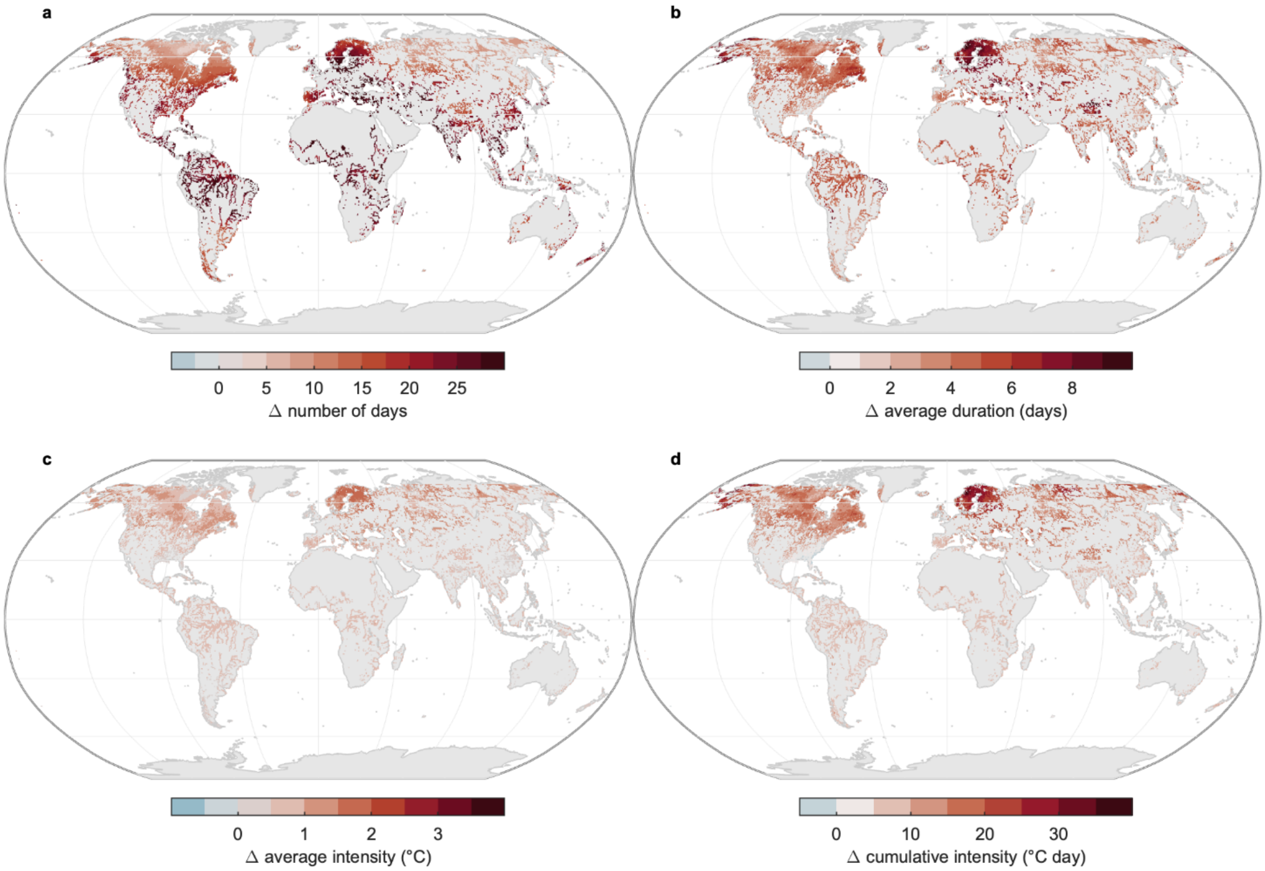

Supplementary Figure 1. Spatial differences in variability of lake surface heatwaves under climate change. Shown are the variability across studied sites in terms of temporal differences (2000-2022 relative to 1980-1999) in the (**a**) occurrence, (**b**) average duration, (**c**) average intensity, and (**d**) cumulative intensity of lake surface heatwaves across the studied lakes.

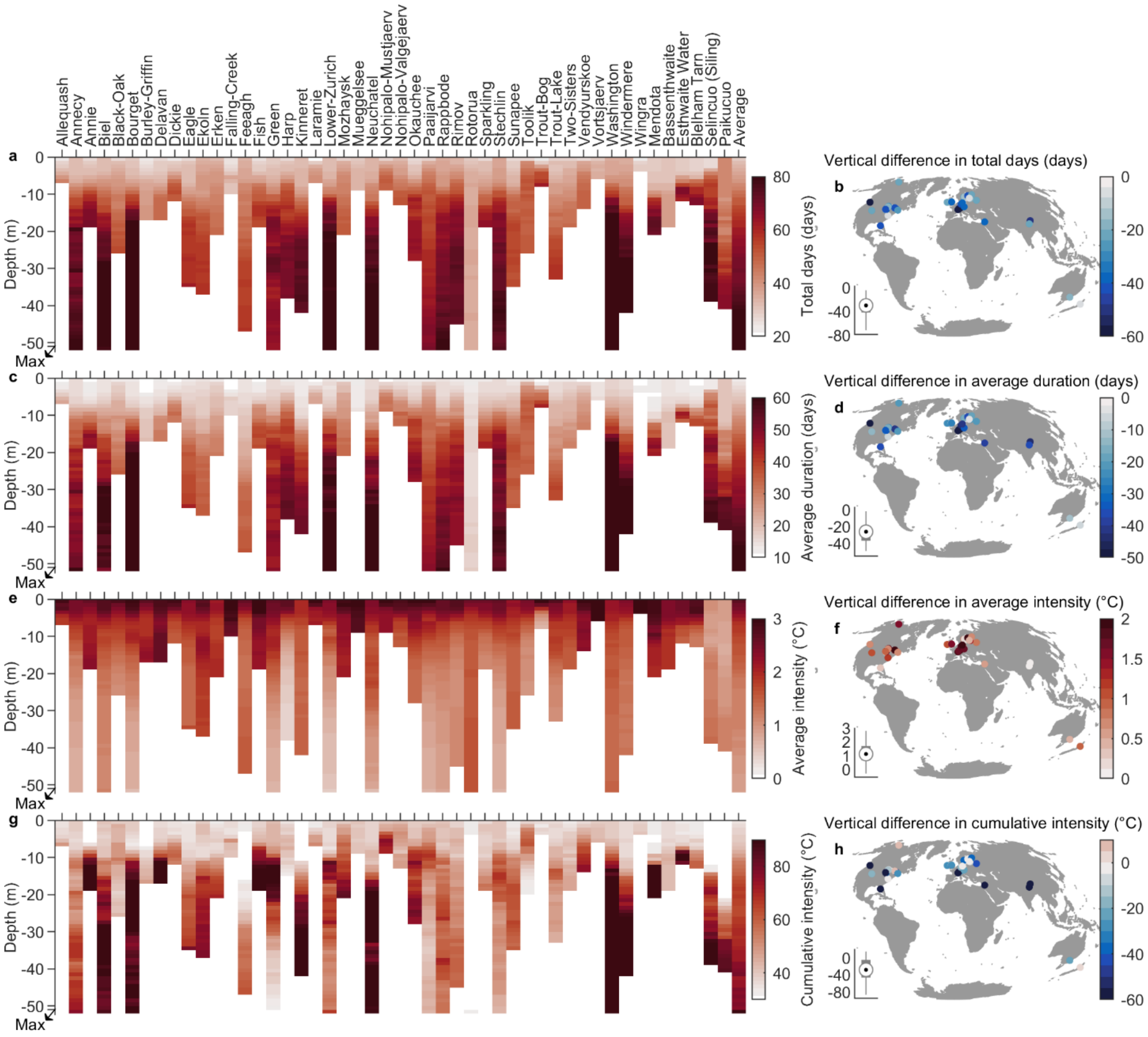

Supplementary Figure 2. Surface and subsurface heatwaves in 48 individual lakes. Heatwave metrics include **(a, b)** total days (unit: days), **(c, d)** average duration (unit: days), **(e, f)** average intensity (unit: °C), and **(g, h)** cumulative intensity (unit: °C). Panels **(a, c, e, g)** are profiles of annual means during the period of 1981-2020. Vertical distributions are displayed per meter, and for depths larger than 50 meters, only results for the maximum depth are shown. Panels **(b, d, f, h)** show the spatial distribution of vertical differences (i.e., difference between lake surface and bottom), with their statistics displayed as boxplots in each panel. Lake basic information corresponds to Supplementary Table 1.

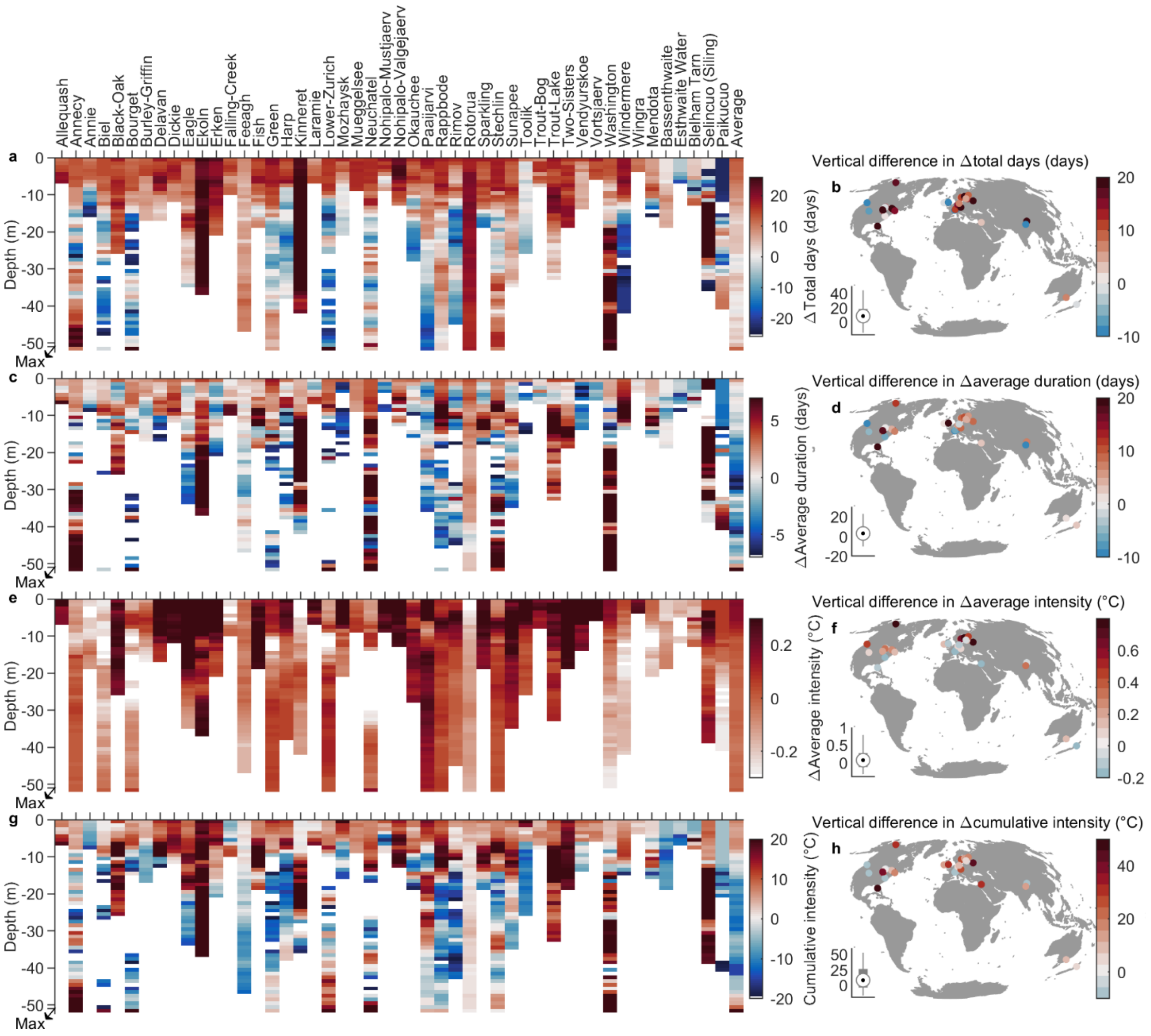

Supplementary Figure 3. Evolution of surface and subsurface heatwaves in 48 individual lakes. Heatwave metrics include **(a, b)** total days (unit: days), **(c, d)** average duration (unit: days), **(e, f)** average intensity (unit: °C), and **(g, h)** cumulative intensity (unit: °C). Panels **(a, c, e, g)** are profiles of temporal changes during the first half (1981-2000) and second half (2001-2020) periods. Vertical distributions are displayed per meter, and for depths larger than 50 meters, only results for the maximum depth are shown. Panels **(b, d, f, h)** show the spatial distribution of vertical differences (i.e., difference between lake surface and bottom), with their statistics displayed as boxplots in each panel. Lake basic information corresponds to Supplementary Table 1.

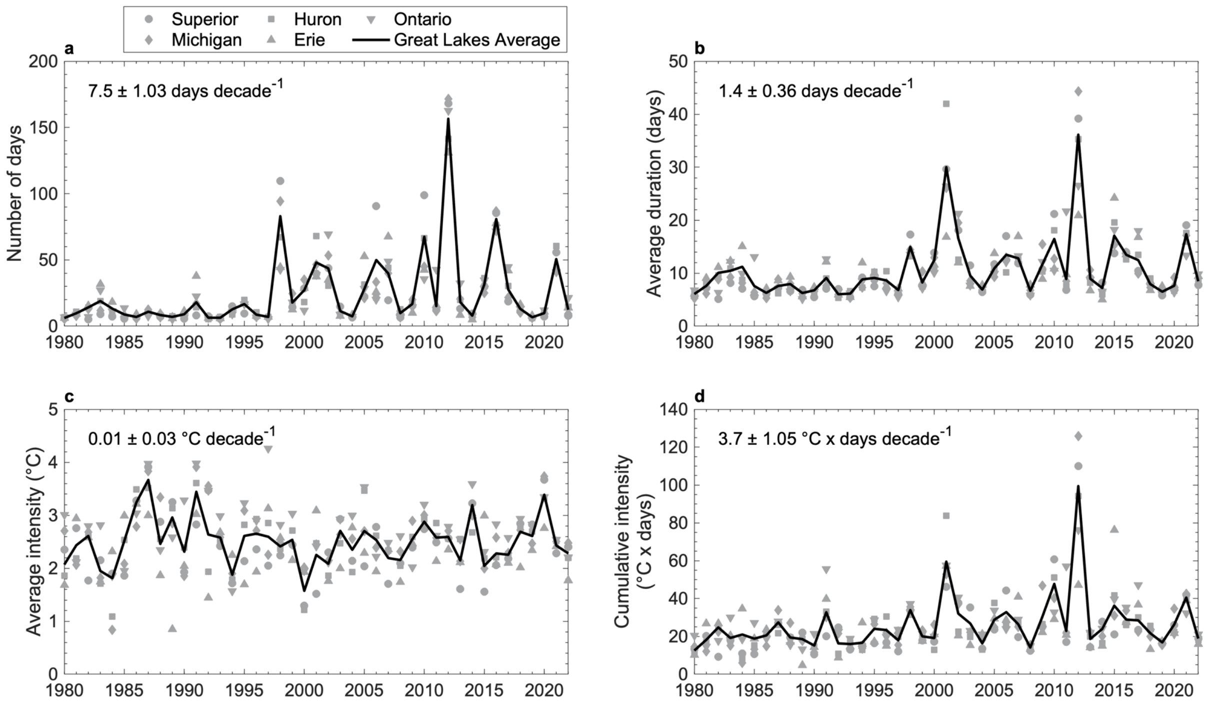

Supplementary Figure 4. Evolution of surface heatwave in the Great Lakes. Shown are the simulated changes in the (**a**) occurrence, (**b**) average duration, (**c**) average intensity, and (**d**) average cumulative intensity of lake surface heatwaves from 1980 to 2022 in the Great Lakes.

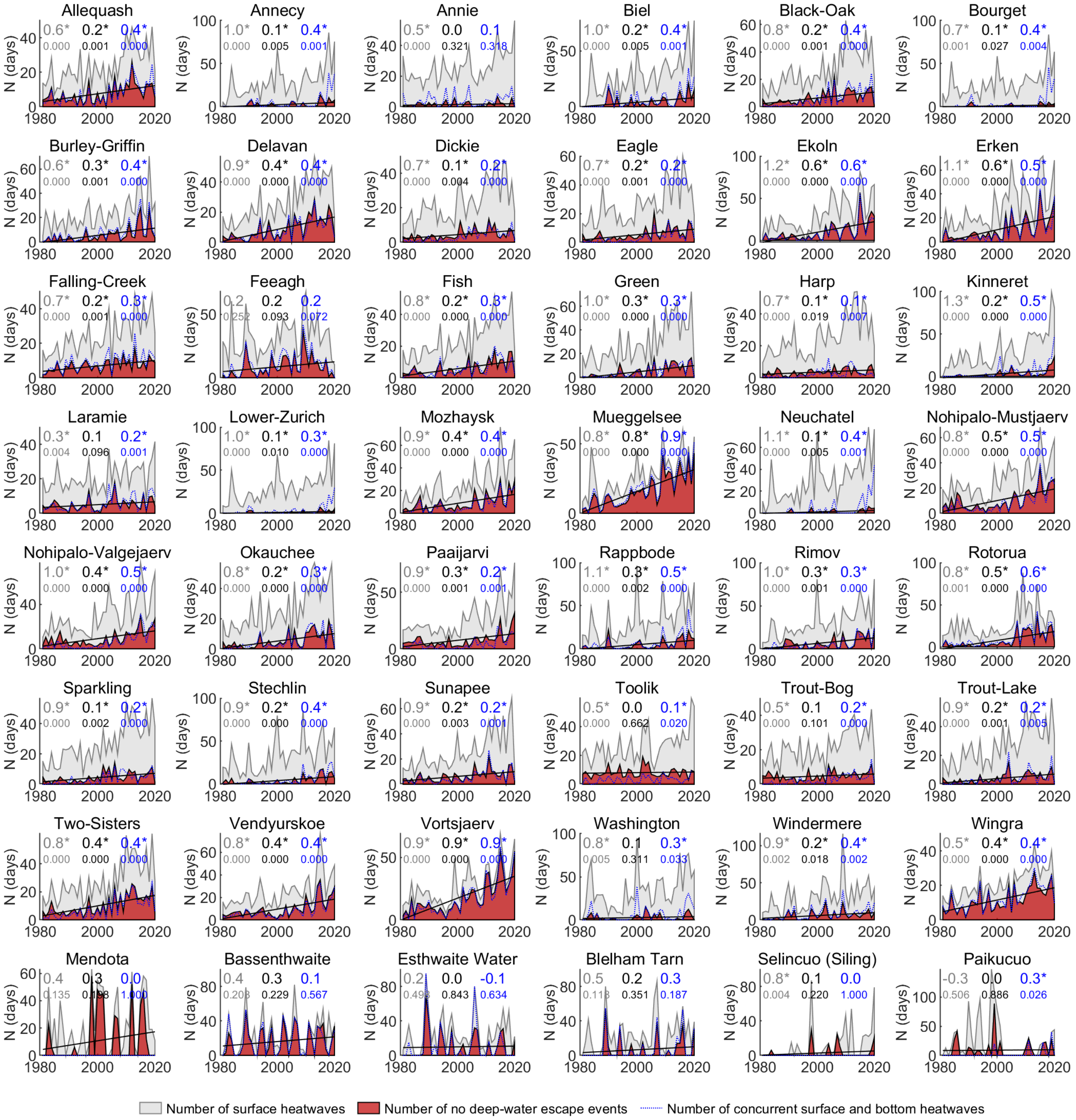

Supplementary Figure 5. Evolution of total surface heatwave days without thermal escape in deeper water in 48 individual lakes, shown as black lines (with red shading). Linear fitting lines for the surface heatwave days without escape depth are also shown in each panel, with the slope (unit: days/year) and statistical significance level (P<0.05) indicated by black text and an asterisk (*). Also shown are total number of surface heatwaves (dark-gray lines with gray shading) and concurrent lake surface and bottom heatwaves (blue dotted lines), with their respective linear fitting slopes (unit: days/year) and significance levels (P<0.05) displayed in gray and blue text. The lake names are featured in the title of the respective panel. The significance level of the linear trend was tested using a one-sided t-test on the slope coefficient. The exact P values are also shown below the slope values. No adjustments were made for multiple comparisons.

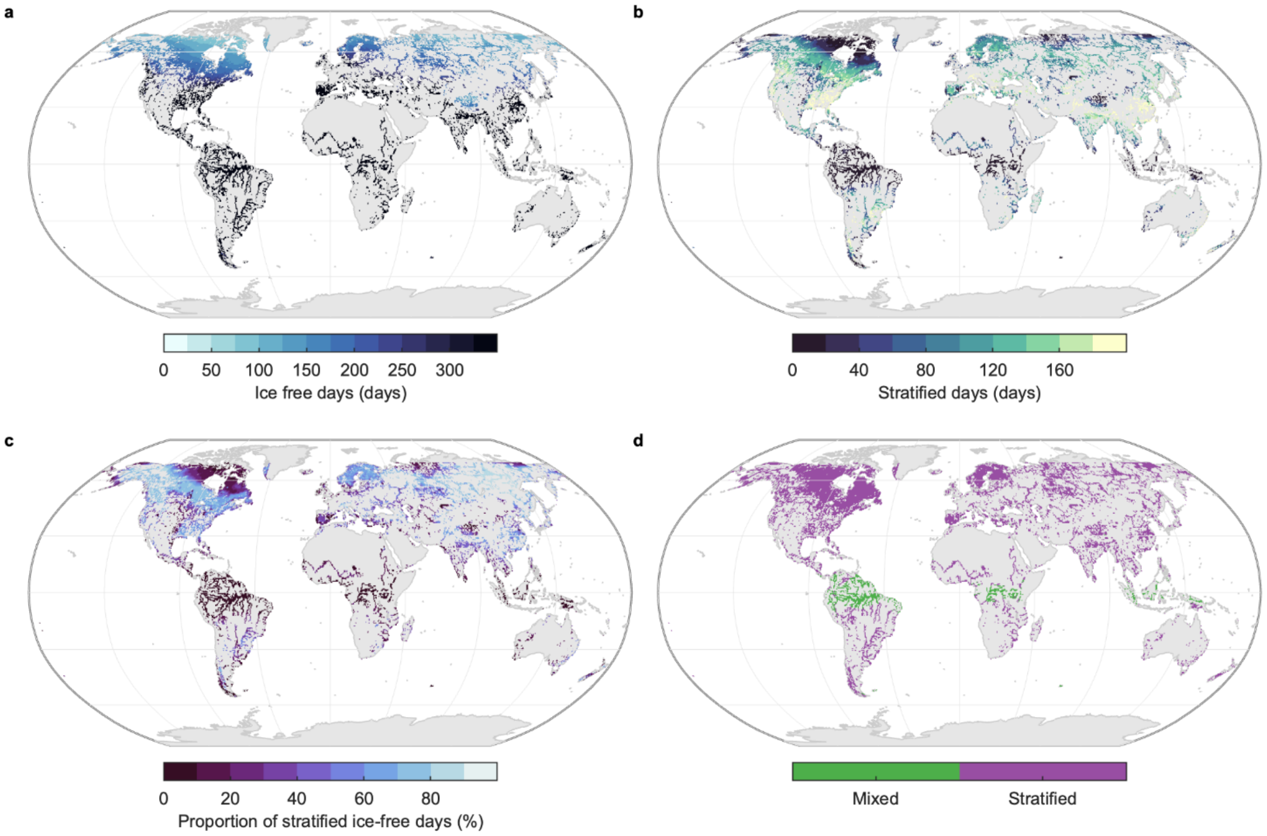

Supplementary Figure 6. Shown, for the historic period (1980-2022), is the (**a**) the average count of ice-free days (**b**) the number of stratified days, and (**c**) the proportion of stratified ice-free days across the representative lakes investigated in this study. (**d**) A map showing which lakes are categorised as mixed and which ones are stratified worldwide. We define a lake as stratified if there is a temperature difference greater than 1°C between the surface and the bottom water.

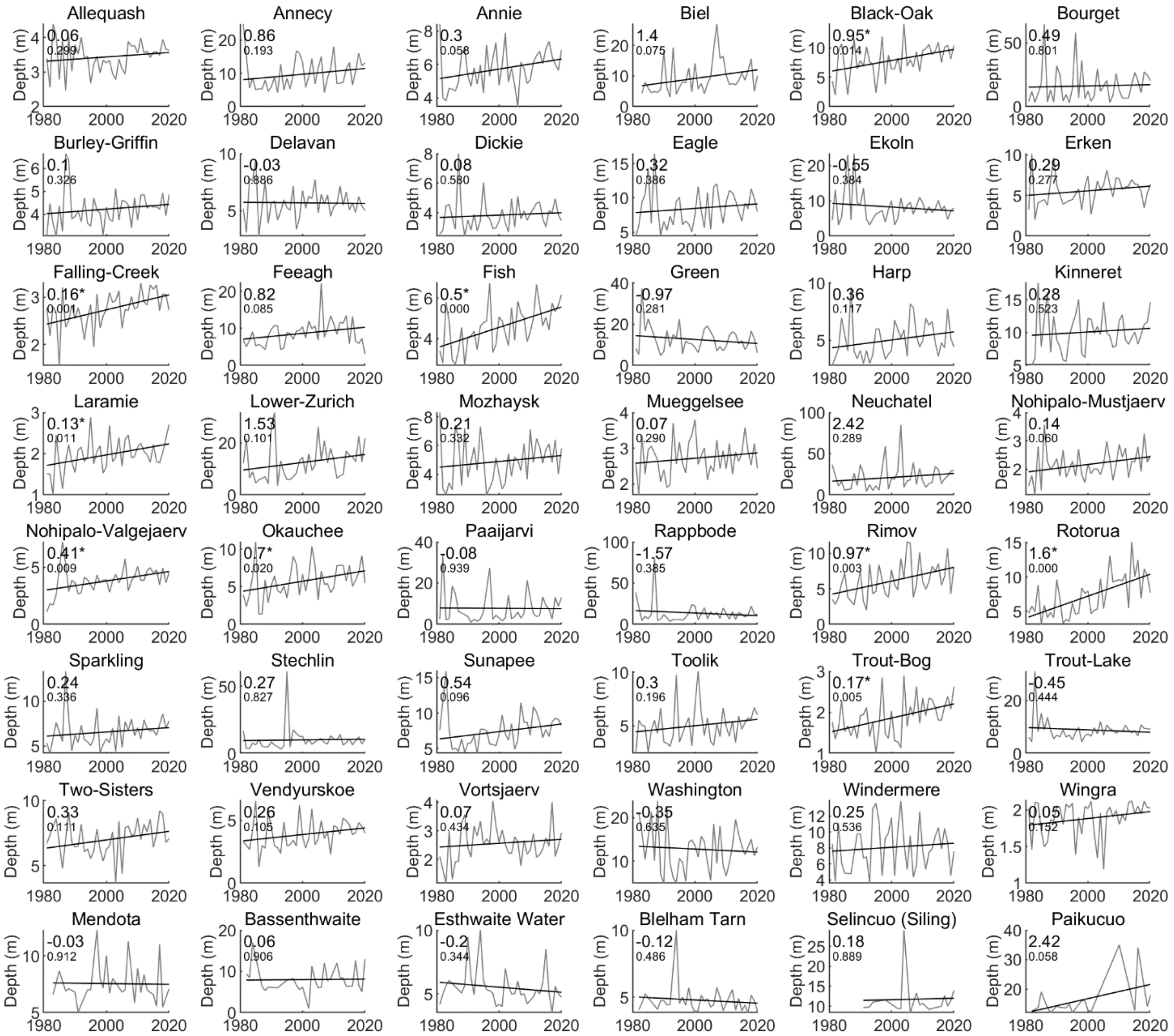

Supplementary Figure 7. Evolution of escape depth for surface aquatic species in 48 individual lakes. Each panel illustrates the annual time series of averaged escape depth for a specific lake, with the lake name featured in the title of the respective panel. Linear fitting lines are also shown in each panel, with the slope (unit: days/decade) and statistical significance level ($P<0.05$) shown in black text and marked with an asterisk (*). The significance level of the linear trend was tested using a one-sided t-test on the slope coefficient. The exact P values are also shown below the slope values. No adjustments were made for multiple comparisons.

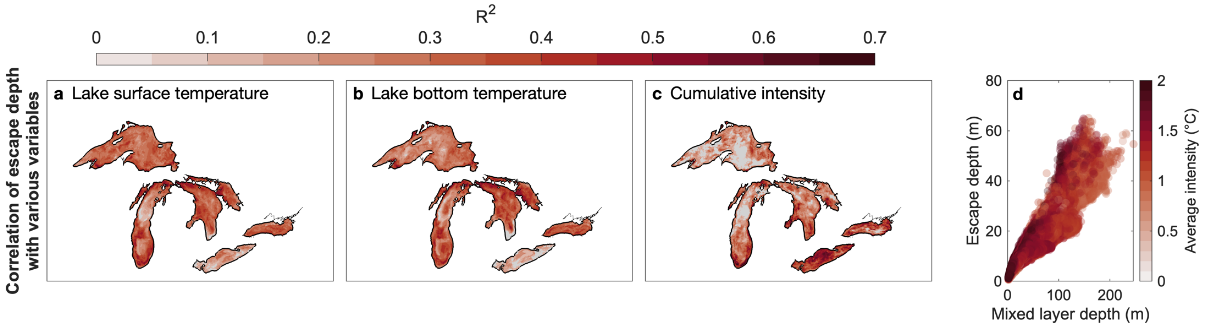

Supplementary Figure 8. Correlation (R^2) between escape depth and lake temperature and heatwave intensity. Correlation between inter-annual variability in escape depth and lake surface temperature **(a)**, lake bottom temperature **(b)**, and average cumulative heatwave intensity **(c)**. The relationship between the average (1980-2022) escape depth, the average intensity of lake surface heatwaves, and the depth of the mixed layer in each Great Lakes grid point **(d)**.

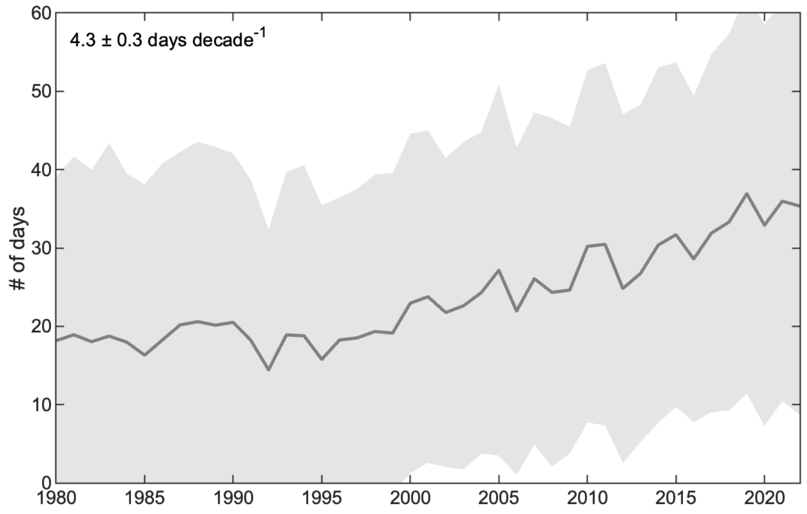

Supplementary Figure 9. Lake bottom heatwave without a lake surface heatwave. Shown are the simulated changes in the average occurrence of lake bottom heatwaves without the presence of a lake surface heatwave from 1980 to 2022. The solid line represents the global average, and the shaded region represents the standard deviation. Trends for the global average time series are shown in the upper left corner of each panel.

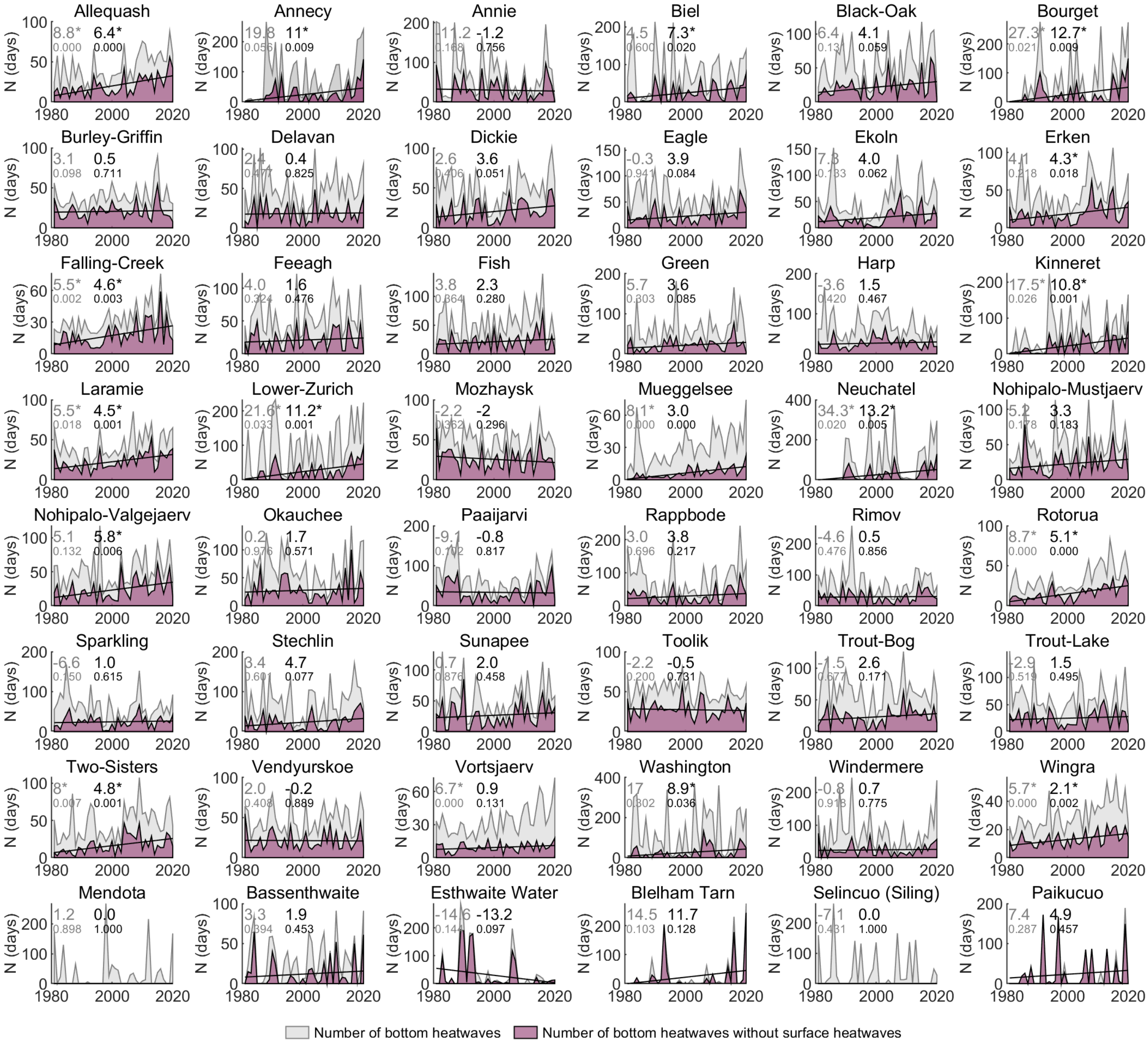

Supplementary Figure 10. Evolution of total bottom heatwave days without surface heatwaves in 48 individual lakes, shown as black lines (with purple shading). Linear fitting lines for the bottom heatwave days without surface heatwaves are also shown in each panel, with the slope (unit: days/decade) and statistical significance level (P<0.05) indicated by black text and an asterisk (*). Also shown are total number of bottom heatwaves (dark-gray lines with gray shading), with the linear fitting slope (unit: days/ decade) and significance levels (P<0.05) displayed in gray text and an asterisk (*). The lake names are featured in the title of the respective panel. The significance level of the linear trend was tested using a one-sided t-test on the slope coefficient. The exact P values are also shown below the slope values. No adjustments were made for multiple comparisons.

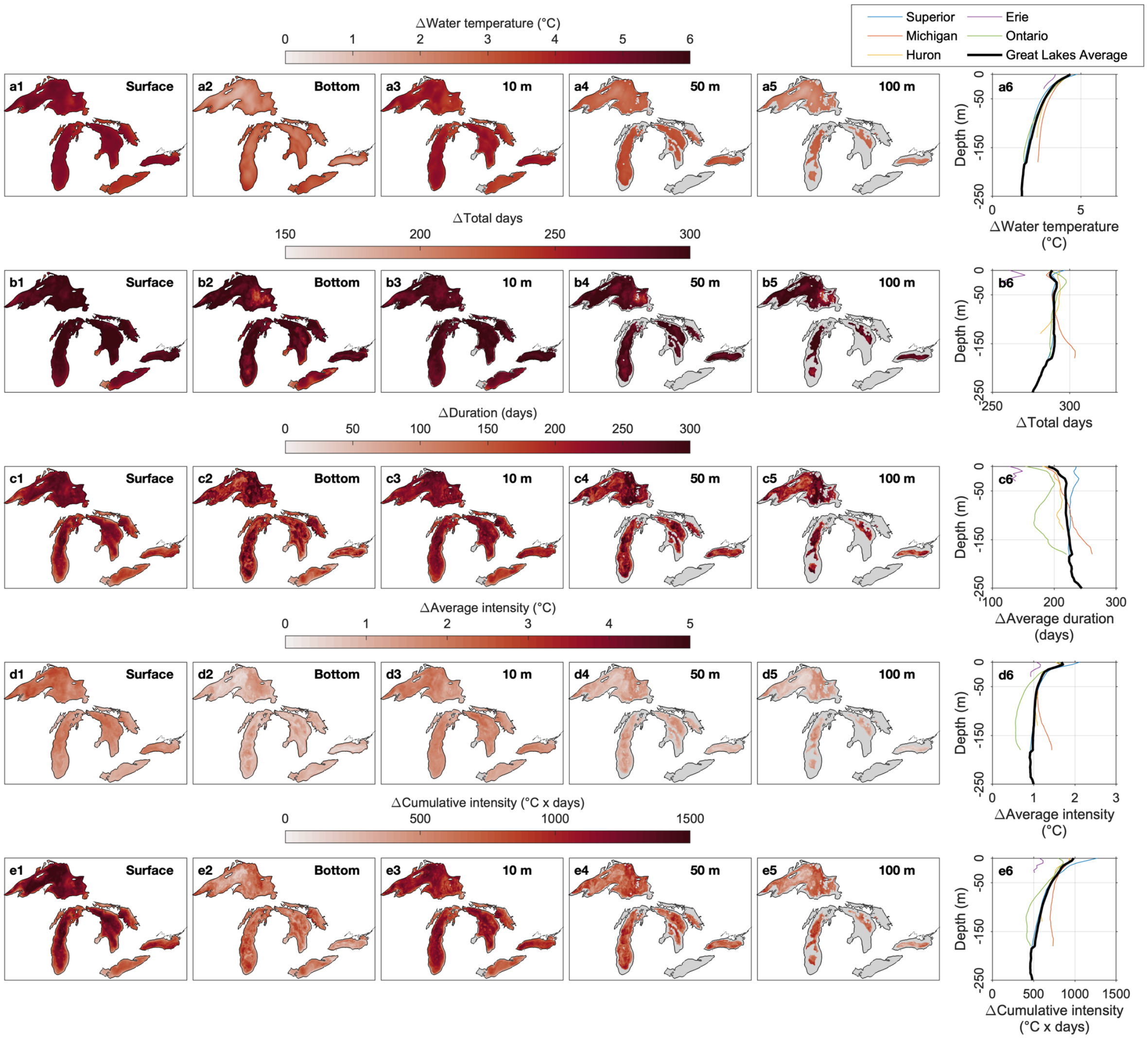

Supplementary Figure 11. Future changes in subsurface heatwaves in the Great Lakes. Shown are the spatial variability of future changes under RCP 8.5 across the Great Lakes at surface, bottom, 10 m, 50 m, and 100 m depths for **(a1-a5)** average water temperature, **(b1-b5)** average occurrence, **(c1-c5)** average duration, **(d1-d5)** average intensity, and **(e1-e5)** average cumulative intensity of lake heatwaves. We also show a lake-wide averaged change in **(a6)** water temperature, **(b6)** occurrence, **(c6)** average duration, **(d6)** average intensity, and **(e6)** cumulative intensity of lake heatwaves with depth. The values are the changes in annual means during 2080-2099 relative to 1980-2022.

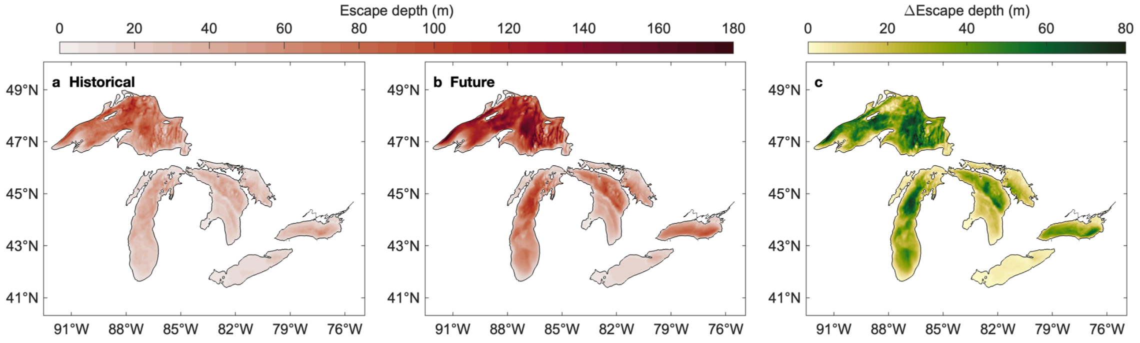

Supplementary Figure 12. Future changes in the escape depth in the Great Lakes. Shown are the spatial variability of average escape depth across the Great Lakes for the **(a)** historical period (1980-2022) and **(b)** future period (2080-2099) under RCP 8.5. The future changes in escape depth relative to the historical period is shown in panel **(c)**.

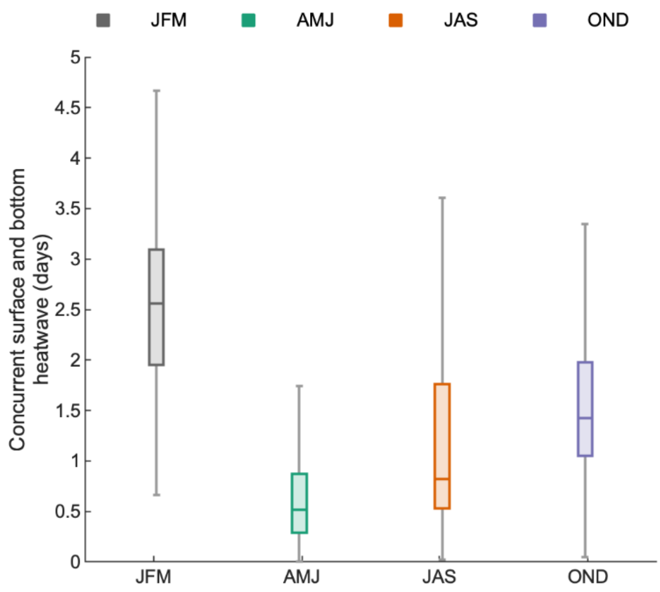

Supplementary Figure 13. Seasonal variability in the average occurrence of vertically compounding heatwaves in lakes during the historic period (1980-2022). In each box plot (n = 16,455), the central mark indicates the median, and the bottom and top edges of the boxes indicate the 25th and 75th percentiles, respectively. The whiskers extend to the most extreme data points not considered outliers.

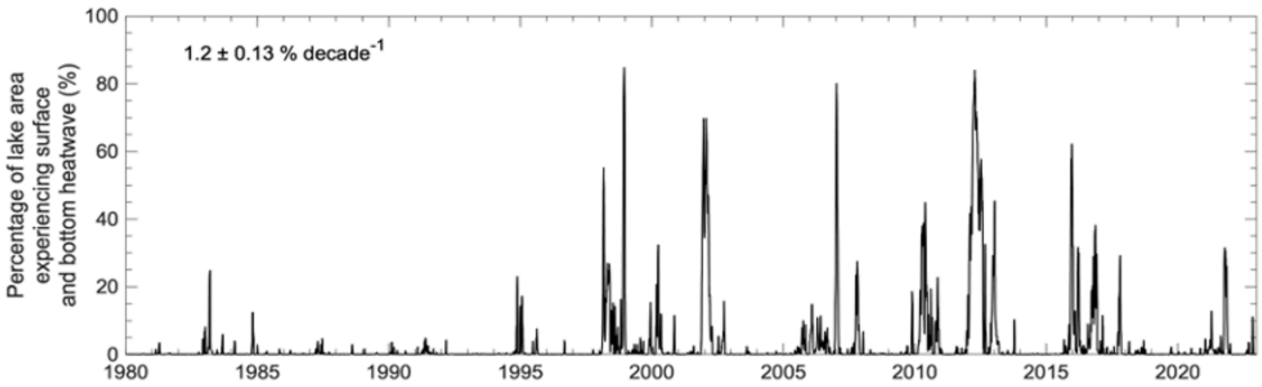

Supplementary Figure 14. Concurrent heatwaves in lake surface and bottom waters in the Great Lakes. Shown are temporal changes in the percentage of the Great Lakes area experiencing a co-occurrence of lake surface and bottom heatwaves.

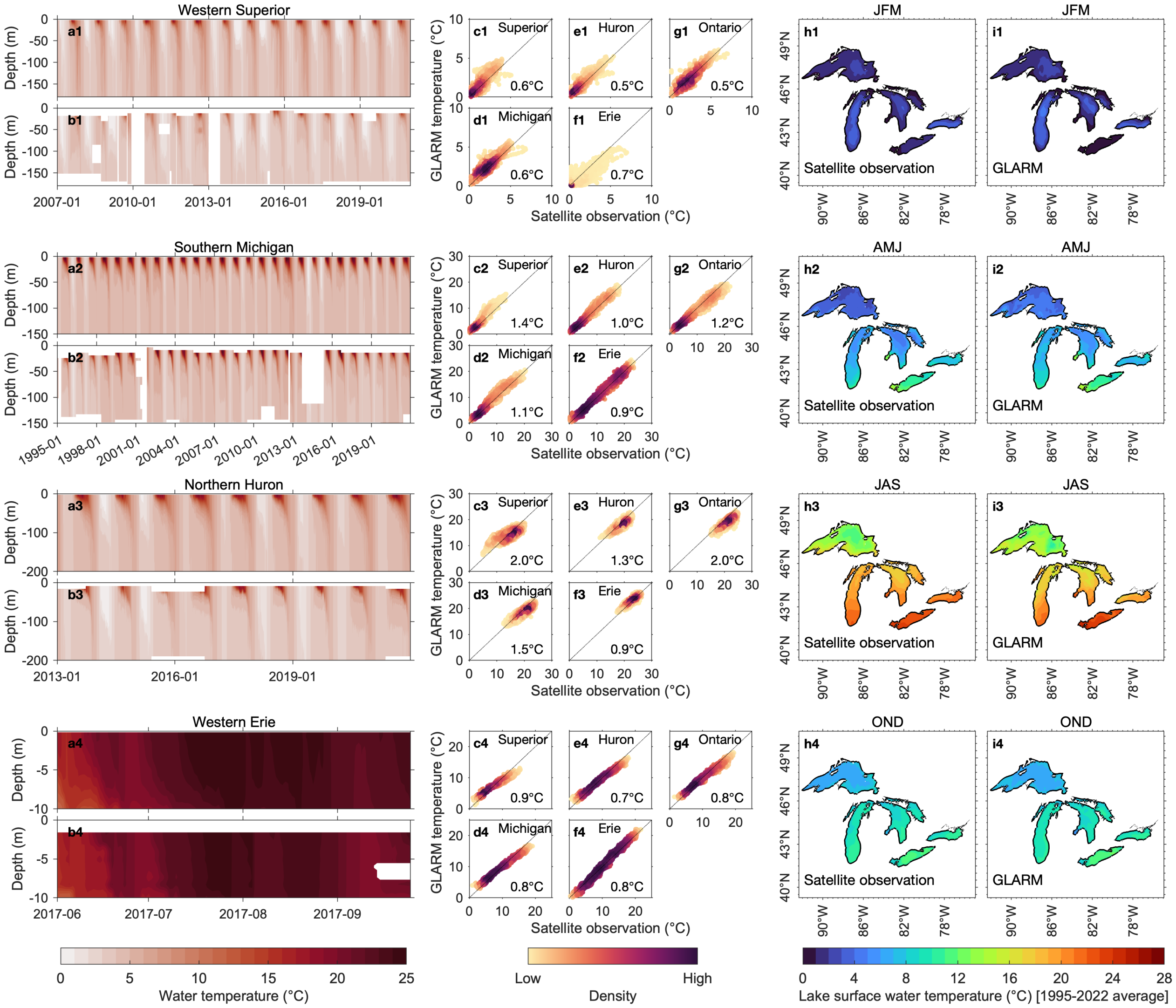

Supplementary Figure 15. Accuracy performance of GLARM-simulated Great Lakes temperature. Comparison of GLARM-simulated daily thermal structure **(a1-a4)** against mooring observations **(b1-b4)**. Panels **(c-g)** represent comparisons between daily GLARM-simulated lakewide average surface temperature and satellite-derived lakewide average surface temperature for each lake. The root-mean-square-error of GLARM-simulated temperature different depths are annotated in each panel. Panels **(h-i)** spatially compare the seasonal climatology of satellite-derived lake surface temperature **(h)** and GLARM-simulated lake surface temperature **(i)**. Panels **(c1-i1)** present winter temperature, **(c2-i2)** present spring temperature, **(c3-i3)** present summer temperature, and **(c4-i4)** present fall temperature.

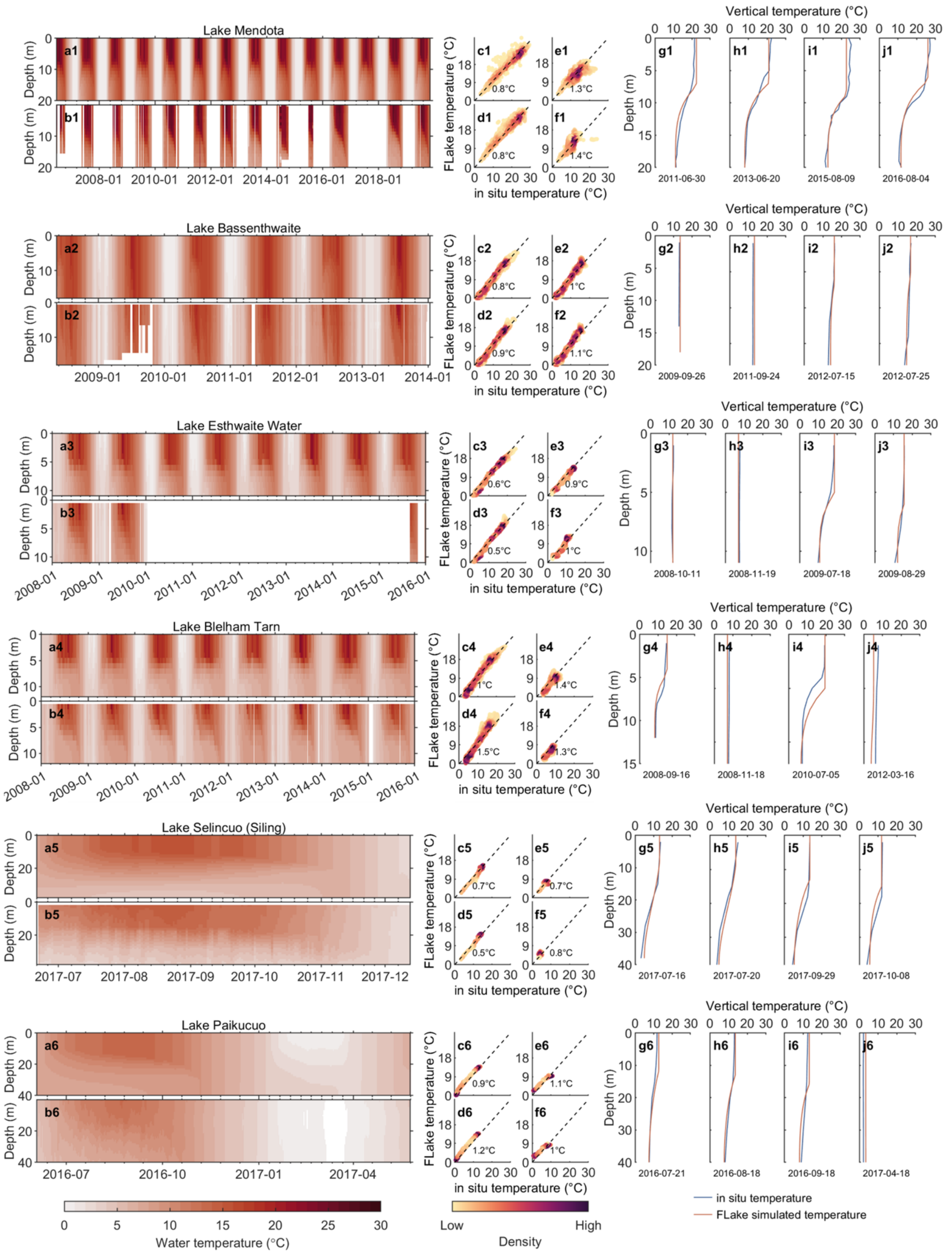

Supplementary Figure 16. Accuracy performance of lake-specific FLake models. The studied lakes include **(a1-j1)** Mendota, **(a2-j2)** Bassenthwaite, **(a3-j3)** Esthwaite Water, **(a4-j4)** Blelham Tarn, **(a5-j5)** Selincuo (Siling), and **(a6-j6)** Paikucuo lakes. Panels **(a)** and **(b)** are daily lake temperature profiles of FLake-calibrated simulations and in situ observations, respectively. Temperature profiles colored in white represent data gaps of in situ data. Panels **(c-f)** represent comparisons between FLake simulated temperature and in situ temperature at four depths, including **(c)** surface, **(d)** 1/3 total depth, **(e)** 2/3 total depth, and **(f)** bottom. Median absolute error of FLake simulations at different depths are also annotated in each panel. Panels **(g-j)** illustrate the

comparison of vertical profiles between FLake simulations and in situ observations over randomly selected four days.

Supplementary Table 1. Basic information of the studied individual lakes, including lake ID, lake name, country, location, maximum depth, and maximum depth with simulations. The vertical temperature data for the initial 42 lakes (Lake IDs 1 to 42) are sourced from ISIMIP2b local simulations, while the temperature profiles for the subsequent 6 lakes (Lake IDs 43 to 48) are simulated using individually calibrated FLake models specific to each lake in this study. Source links, temporal coverages and depths sampled for hourly in situ observations pertaining to the 6 lakes provided in the footnotes below the table.

Lake ID	Lake name	Country	Latitude (°)	Longitude (°)	Max depth of sampling site (m)	Max depth with simulations (m)
1	Allequash	USA	46.04	-89.62	8	6
2	Annecy	France	45.87	6.17	65	61
3	Annie	USA	27.21	-81.35	68	18
4	Biel	Switzerland	47.08	7.16	74	71
5	Black-Oak	USA	46.16	-89.32	25.91	25
6	Bourget	France	45.76	5.86	145	141
7	Burley-Griffin	Australia	-35.30	149.07	17	16
8	Delavan	USA	42.61	-88.60	16.46	16
9	Dickie	Canada	45.15	-79.09	12	11
10	Eagle	Canada	44.68	-76.70	31.1	34
11	Ekoln	Sweden	59.75	17.62	50	36
12	Erken	Sweden	59.84	18.63	21	20
13	Falling-Creek	USA	37.31	-79.84	9.3	9
14	Feeagh	Ireland	53.90	-9.50	44	46
15	Fish	USA	43.29	-89.65	18.9	18
16	Green	USA	43.81	-89.00	72	71
17	Harp	Canada	45.38	-79.13	37.5	37
18	Kinneret	Israel	32.49	35.35	45	41
19	Laramie	USA	40.62	-105.84	6.4	6
20	Lower-Zurich	Switzerland	47.28	8.58	136	131
21	Mozhaysk	Russia	55.59	35.82	23	20
22	Mueggelsee	Germany	52.43	13.65	7.7	8
23	Neuchatel	Switzerland	46.54	6.52	152	151
24	Nohipalo-Mustjaerv	Estonia	57.93	27.34	8.9	8
25	Nohipalo-Valgejaerv	Estonia	57.94	27.35	12.5	12
26	Okauchee	USA	43.13	-88.43	28.65	27
27	Paaijarvi	Finland	61.07	25.13	85	71
28	Rappbode	Germany	51.74	10.89	89	81
29	Rimov	Czechia	48.85	14.49	44	44
30	Rotorua	New Zealand	-38.08	176.28	52.9	51
31	Sparkling	USA	46.01	-89.70	20	18
32	Stechlin	Germany	53.17	13.03	69.5	61
33	Sunapee	USA	43.23	-72.50	34	34
34	Toolik	USA	68.63	-149.60	/	25
35	Trout-Bog	USA	46.04	-89.69	7.9	7
36	Trout-Lake	USA	46.03	-89.67	35.7	32
37	Two-Sisters	USA	45.77	-89.53	19.2	18
38	Vendyurskoe	Russia	62.10	33.10	13.4	13
39	Vortsjaerv	Estonia	58.31	26.01	6	5
40	Washington	USA	47.64	-122.27	65.2	61
41	Windermere	United Kingdom	54.31	-2.95	64	41

42	Wingra	USA	43.05	-89.43	6.7	3
43	Mendota Lake	USA	43.10	-89.41	/	20
44	Bassenthwaite Lake	UK	54.66	-3.22	/	18
45	Esthwaite Water	UK	54.36	-2.99	/	11
46	Blelham Tarn	UK	54.40	-2.98	/	12
47	Selincuo (Siling) Lake	China	31.76	88.97	/	38
48	Paikucuo Lake	China	28.83	85.60	42	40

Note:

Mendota Lake: https://lter.limnology.wisc.edu/, 8-year observations at 23 sampled depths during the period of 2006-2019;

Bassenthwaite Lake: https://eip.ceh.ac.uk/data, 7-year observations at 12 sampled depths during the period of 2008-2014;

Esthwaite Water: https://eip.ceh.ac.uk/data, 5-year observations at 12 sampled depths (0.43-11.43m) during the period of 2008-2016;

Blelham Tarn: https://eip.ceh.ac.uk/data, 9-year observations at 12 sampled depths (0.5-12m) during the period of 2008-2016;

Selincuo (Siling) Lake: https://poles.tpdc.ac.cn/, 0.5-year observations at 5 sampled depths (2-38m) during the year of 2017;

Paikucuo Lake: https://poles.tpdc.ac.cn/, 2-year observations at 7 sampled depths (0-40m) during the year of 2016-2017.

Supplementary Table 2. Annual mean statistics of lake surface and bottom heatwave events during the historical period (1981-2020).

Lake ID	Lake name	Surface total days (days)	Surface average duration (days)	Surface average intensity (°C)	Surface cumulative intensity (°C)	Bottom total days (days)	Bottom average duration (days)	Bottom average intensity (°C)	Bottom cumulative intensity (°C)
1	Allequash	24.0	10.9	2.9	35.0	43.0	24.0	1.8	49.6
2	Annecy	31.7	14.9	2.5	38.7	86.1	63.7	0.8	76.4
3	Annie	27.1	10.6	2.6	27.9	69.0	47.4	2.3	148.5
4	Biel	31.5	14.1	2.8	40.0	77.3	49.4	1.1	85.9
5	Black-Oak	27.0	12.8	2.8	38.7	53.8	36.1	1.0	43.0
6	Bourget	29.6	13.5	3.0	40.0	101.2	80.3	0.6	105.3
7	Burley-Griffin	23.3	9.3	2.7	24.6	40.0	19.7	2.2	47.7
8	Delavan	26.3	11.9	2.8	36.2	48.2	31.0	2.6	101.4
9	Dickie	23.5	11.0	2.9	35.6	50.2	30.6	1.5	54.1
10	Eagle	26.6	12.6	2.3	33.4	61.8	44.1	1.7	79.1
11	Ekoln	33.7	14.0	2.7	39.8	58.2	35.6	1.8	77.7
12	Erken	32.3	13.2	2.1	31.9	48.2	30.3	2.0	70.6
13	Falling-Creek	24.6	9.2	3.7	37.0	31.3	16.4	2.5	40.4
14	Feeagh	34.0	13.6	2.6	33.4	57.3	36.2	1.5	62.8
15	Fish	24.9	11.0	3.3	37.9	53.7	37.7	2.2	92.8
16	Green	28.8	15.0	2.6	42.0	63.5	49.2	0.7	29.3
17	Harp	25.9	12.0	2.7	35.7	68.9	53.0	0.4	21.3
18	Kinneret	32.3	13.3	2.0	26.9	71.8	51.7	1.5	101.1
19	Laramie	20.9	9.6	3.2	33.6	41.2	23.8	2.1	51.9
20	Lower-Zurich	32.4	14.7	2.4	37.3	107.0	88.0	0.4	66.0
21	Mozhaysk	27.4	12.1	2.9	39.0	51.1	33.5	2.1	83.4
22	Mueggelsee	29.1	11.5	3.4	40.6	33.5	13.6	2.7	40.6
23	Neuchatel	33.0	15.6	2.4	37.6	140.0	125.9	0.6	154.3
24	Nohipalo-Mustjaerv	28.4	10.8	3.5	40.7	49.2	31.8	2.2	70.5
25	Nohipalo-Valgejaerv	32.1	12.8	2.9	41.8	51.7	32.9	1.5	47.3
26	Okauchee	25.9	12.6	3.1	39.5	62.3	46.9	1.4	75.3
27	Paaijarvi	28.1	12.9	3.0	39.1	69.4	48.2	0.7	43.0
28	Rappbode	35.1	14.6	2.6	41.0	78.8	59.4	0.8	56.5
29	Rimov	33.0	13.1	2.8	38.2	72.3	47.5	1.1	59.4
30	Rotorua	29.0	11.8	2.6	32.8	35.4	17.2	1.6	31.5
31	Sparkling	26.8	12.6	2.6	36.4	65.7	44.4	1.1	54.3
32	Stechlin	37.0	15.0	2.6	41.4	75.3	55.7	0.7	44.2
33	Sunapee	26.3	12.9	2.2	31.8	54.1	35.1	1.6	60.6
34	Toolik	27.1	13.8	2.5	38.1	50.6	34.7	1.0	29.3
35	Trout-Bog	22.1	9.8	3.5	36.3	62.8	43.9	0.7	26.7
36	Trout-Lake	27.2	12.7	2.7	35.9	58.2	38.1	1.0	45.1
37	Two-Sisters	28.0	12.8	2.2	33.7	47.3	29.2	1.5	46.8
38	Vendyurskoe	26.5	11.1	2.9	36.0	49.5	30.1	2.3	75.4
39	Vortsjaerv	28.9	10.8	3.4	39.3	35.0	14.9	3.0	43.2
40	Washington	35.6	17.1	2.2	39.1	141.9	126.3	1.4	283.7
41	Windermere	35.1	15.4	2.4	36.7	82.2	60.5	1.3	103.4
42	Wingra	21.6	9.0	4.0	37.3	30.0	12.1	3.3	40.4
43	Mendota Lake	29.1	11.7	2.9	32.4	71.4	45.0	2.0	118.8
44	Bassenthwaite Lake	30.6	14.2	2.4	36.4	37.6	19.0	1.9	42.2
45	Esthwaite Water	29.2	13.1	2.5	34.0	60.0	34.1	1.5	80.6
46	Blelham Tarn	28.3	11.0	2.8	31.5	68.3	38.3	1.2	58.9
47	Selincuo (Siling) Lake	26.3	13.1	1.1	14.7	78.0	57.7	1.4	97.8

48	Paikucuo Lake	42.7	19.1	0.9	21.3	66.2	55.5	1.2	84.3

Supplementary Table 3. Changes of lake surface and bottom heatwave events between the period of 1981-2000 and 2001-2020. The "*" after the numbers indicates the temporal changes are statistically significant (*P*<0.05). The significance level of the temporal changes was tested using a one-sided t-test on the linear slope coefficient. The exact *P* values are also shown in parentheses. No adjustments were made for multiple comparisons.

Lake ID	Lake name	Δ Surface total days (days)	Δ Surface average duration (days)	Δ Surface average intensity (℃)	Δ Surface cumulative intensity (℃)	Δ Bottom total days (days)	Δ Bottom average duration (days)	Δ Bottom average intensity (℃)	Δ Bottom cumulative intensity (℃)
1	Allequash	11.1*(0.000)	1.3(0.068)	0.4*(0.002)	7.4*(0.015)	19.9*(0.000)	6.8*(0.032)	0.2(0.159)	18.9*(0.031)
2	Annecy	13.2*(0.000)	2.0 (0.078)	-0.1(0.676)	4.3(0.243)	10.4(0.217)	6.7(0.243)	0.0 (0.612)	19.7(0.181)
3	Annie	9.4*(0.000)	0.5(0.114)	-0.2*(0.009)	-3.6(0.73)	-26.9(0.168)	-19.7(0.100)	-0.1(0.27)	-91.6(0.055)
4	Biel	14.0*(0.000)	1.0 (0.369)	-0.3(0.140)	0.4(0.948)	-1.9(0.985)	-0.9(0.823)	0.0 (0.701)	17.0 (0.729)
5	Black-Oak	18.0*(0.000)	3.3*(0.002)	0.2(0.299)	11.6*(0.011)	13.7(0.131)	6.0 (0.528)	0.2*(0.02)	15.1(0.266)
6	Bourget	10.9*(0.001)	1.7(0.164)	-0.2(0.327)	4.3(0.355)	43.4(0.115)	32.8(0.056)	0.1*(0.048)	76.6*(0.014)
7	Burley-Griffin	12.1*(0.000)	2.1*(0.017)	-0.1(0.551)	4.0 (0.070)	5.5(0.098)	0.3(0.845)	-0.3*(0.001)	-6.4(0.694)
8	Delavan	17.9*(0.000)	2.8*(0.006)	0.4*(0.001)	13.0*(0.001)	4.7(0.477)	-5.8(0.306)	0.1(0.749)	-24.1(0.339)
9	Dickie	14.7*(0.000)	3.2*(0.009)	0.5*(0.005)	15.2*(0.003)	8.4(0.406)	0.6(0.802)	0.3*(0.002)	1.2(0.627)
10	Eagle	16.3*(0.000)	2.4(0.070)	0.3(0.079)	9.0 (0.086)	-4.7(0.941)	-9.8(0.345)	-0.1(0.222)	-21.0 (0.451)
11	Ekoln	25.4*(0.000)	4.1*(0.007)	0.6*(0.012)	19.8*(0.001)	22.9(0.152)	6.2(0.961)	0.3(0.133)	29.0 (0.657)
12	Erken	23.7*(0.000)	3.0 (0.052)	0.7*(0.000)	17.6*(0.001)	12.0 (0.218)	-5.9(0.057)	0.0 (0.394)	-7.0 (0.351)
13	Falling-Creek	12.9*(0.000)	0.3(0.354)	-0.2(0.232)	-4.7(0.903)	12.5*(0.002)	6.3*(0.043)	0.0 (0.670)	11.4(0.111)
14	Feeagh	8.7(0.252)	1.3(0.906)	0.0 (0.608)	2.3(0.832)	9.7(0.583)	-0.5(0.989)	-0.2(0.216)	-12.4(0.297)
15	Fish	15.9*(0.000)	2.0*(0.031)	0.2(0.054)	7.8*(0.014)	9.5(0.364)	3.6(0.981)	0.3*(0.003)	12(0.794)
16	Green	20.4*(0.000)	5.0*(0.002)	0.1(0.323)	13.7*(0.014)	7.3(0.303)	-0.9(0.619)	0.0 (0.203)	3.6(0.292)
17	Harp	16.2*(0.000)	2.9(0.059)	0.3*(0.043)	11.9*(0.025)	-2.9(0.42)	-0.9(0.52)	0.1(0.107)	2.9(0.827)
18	Kinneret	24.3*(0.000)	1.5(0.155)	-0.3*(0.002)	-0.3(0.593)	21.7(0.111)	-1.2(0.457)	-0.1(0.437)	-31.7(0.658)
19	Laramie	6.2*(0.004)	-0.1(0.514)	0.3(0.105)	1.9(0.965)	13.9*(0.018)	4.1(0.228)	0.2(0.119)	16.3(0.276)
20	Lower-Zurich	15.8*(0.000)	3.2*(0.003)	-0.1(0.788)	5.8(0.082)	28.6(0.098)	23.4(0.057)	0.0 (0.325)	58.7*(0.02)
21	Mozhaysk	17.5*(0.000)	3.5*(0.000)	0.9*(0.000)	20.9*(0.000)	-2.6(0.362)	-5.9(0.094)	-0.1(0.706)	-24.1(0.095)
22	Mueggelsee	15.3*(0.000)	2.5*(0.004)	0.0 (0.428)	7.0*(0.031)	16.0*(0.000)	1.9(0.157)	0.1(0.228)	6.0 (0.138)
23	Neuchatel	16.3*(0.000)	3.6*(0.024)	-0.1(0.931)	7.2(0.097)	5.5(0.689)	9.4(0.606)	0.2(0.064)	58.3*(0.027)
24	Nohipalo-Mustjaerv	18.5*(0.000)	1.6(0.081)	0.7*(0.008)	10.3*(0.007)	7.3(0.178)	-5.1(0.49)	0.2*(0.002)	1.7(0.464)
25	Nohipalo-Valgejaerv	22.3*(0.000)	3.0*(0.010)	0.4*(0.006)	14.1*(0.005)	10.2(0.132)	-0.6(0.808)	0.3(0.105)	16.6(0.301)
26	Okauchee	16.6*(0.000)	3.0*(0.004)	0.0 (0.415)	8.1*(0.015)	-11.6(0.976)	-20.0 (0.191)	0.2*(0.039)	-20.8(0.615)
27	Paaijarvi	19.2*(0.000)	2.3(0.074)	0.9*(0.009)	16.9*(0.002)	-18.7(0.102)	-8.2(0.141)	0.1(0.299)	4.4(0.719)
28	Rappbode	17.7*(0.000)	4.0*(0.003)	0.1(0.347)	11.5*(0.008)	2.5(0.696)	-1.2(0.706)	0.0 (0.079)	4.8(0.227)

29	Rimov	16.5*(0.000)	2.8*(0.009)	0.1(0.683)	6.7*(0.033)	-14.1(0.476)	-6.1(0.532)	0.0 (0.323)	-21.5(0.205)
30	Rotorua	17.9*(0.001)	3.9*(0.003)	-0.5*(0.000)	7.4(0.374)	17.9*(0.000)	1.2(0.852)	-0.2*(0.008)	0.6(0.644)
31	Sparkling	17.9*(0.000)	2.4*(0.010)	0.3*(0.013)	8.2*(0.030)	-14.9(0.150)	-7.4(0.172)	0.2*(0.019)	-9.7(0.119)
32	Stechlin	17.5*(0.000)	3.0*(0.034)	-0.1(0.603)	7.6(0.074)	10.2(0.601)	4.5(0.852)	0(0.388)	-0.2(0.716)
33	Sunapee	16.5*(0.000)	2.3*(0.009)	0.3*(0.008)	9.2*(0.007)	1.5(0.876)	-4.8(0.277)	0.1(0.804)	-6.8(0.186)
34	Toolik	9.8*(0.000)	2.5*(0.006)	0.9*(0.000)	22.3*(0.000)	-7.6(0.200)	-8.6*(0.004)	0(0.390)	-9.3(0.079)
35	Trout-Bog	9.0*(0.000)	0.6(0.130)	0.2*(0.031)	4.0*(0.039)	3.2(0.677)	-7.2(0.118)	0.1*(0.017)	0.7(0.638)
36	Trout-Lake	18.4*(0.000)	3.9*(0.005)	0.2(0.107)	11.6*(0.008)	2.9(0.519)	3.2(0.756)	0.1(0.509)	15.3(0.761)
37	Two-Sisters	16.3*(0.000)	3.9*(0.001)	0.6*(0.001)	17.6*(0.001)	18.3*(0.007)	4.4(0.371)	0.3*(0.016)	17.6(0.075)
38	Vendyurskoe	15.4*(0.000)	1.7*(0.022)	0.7*(0.006)	9.9*(0.010)	6.1(0.408)	-3.7(0.175)	0.2(0.136)	-6.8(0.565)
39	Vortsjaerv	18.2*(0.000)	1.3(0.188)	0.4*(0.033)	9.5*(0.025)	13.3*(0.000)	-2.5*(0.038)	0.5*(0.002)	6.5(0.195)
40	Washington	13.5*(0.013)	1.6(0.732)	0.2(0.115)	3.5(0.994)	28.7(0.765)	26.5(0.778)	-0.3*(0.012)	97.7(0.529)
41	Windermere	19.2*(0.004)	3.7(0.325)	0.1(0.948)	5(0.790)	-22.1(0.418)	-22.6(0.188)	-0.2*(0.008)	-69.2*(0.020)
42	Wingra	10.7*(0.000)	0.6(0.118)	0.2*(0.026)	4.2*(0.034)	11.7*(0.000)	0.4(0.448)	0.3*(0.006)	3.1(0.104)
43	Mendota Lake	7.1(0.468)	1.5(0.917)	0.0 (0.734)	2.0 (0.861)	-37.3(0.586)	-16.7(0.588)	-0.2(0.885)	-37.0 (0.746)
44	Bassenthwaite Lake	1.5(0.763)	0.1(0.920)	-0.2(0.389)	-4.7(0.667)	5.7(0.752)	-0.3(0.566)	-0.1(0.165)	-8.6(0.167)
45	Esthwaite Water	-4.0 (0.932)	-1.1(0.686)	-0.2(0.249)	-3.5(0.656)	-45.9(0.09)	-16.5(0.309)	-0.2(0.069)	-56.5(0.152)
46	Blelham Tarn	3.4(0.598)	-2.1(0.530)	-0.1(0.273)	-5.9(0.463)	12.6(0.299)	-27.7(0.56)	0.0 (0.835)	-37(0.673)
47	Selincuo (Siling) Lake	1.4(0.142)	7.0 (0.146)	0.1(0.128)	8.6(0.168)	-25.5(0.437)	-0.4(0.96)	0.1(0.461)	18.0 (0.945)
48	Paikucuo Lake	-23.4(0.719)	-3.7(0.813)	0.1(0.335)	-4.2(0.745)	10.9(0.402)	6.4(0.47)	-0.3(0.299)	-18.5(0.757)

Supplementary Table 4. Annual mean number of lake surface heatwaves without a vertical escape during the period of 1981-2020 and their changes between the period of 1981-2000 and 2001-2020. Annual mean of proportion of no-escape heatwave days relative to total heatwave days and their changes are also presented. The "*" after the numbers indicates the temporal changes are statistically significant (P<0.05). The significance level of the temporal changes was tested using a one-sided t-test on the linear slope coefficient. The exact P values are also shown in parentheses. No adjustments were made for multiple comparisons.

Lake ID	Lake name	number of lake surface heatwaves without a vertical escape (days)	Δ number of lake surface heatwaves without a vertical escape (days)	% of number of lake surface heatwaves without a vertical escape relative to total days of surface heatwaves (%)	Δ % of number of lake surface heatwaves without a vertical escape relative to total days of surface heatwaves (%)
1	Allequash	7.6	5.4*(0.001)	30.2	6.9(0.423)
2	Annecy	2.1	1.7*(0.005)	6.1	2.0 (0.128)
3	Annie	1.5	0.1(0.321)	5.6	-2.1(0.810)
4	Biel	4.1	2.9*(0.005)	12.0	2.6(0.203)
5	Black-Oak	6.4	5.1*(0.001)	23.7	2.3(0.829)
6	Bourget	0.9	0.8*(0.027)	3.0	1.3(0.215)
7	Burley-Griffin	5.5	5.9*(0.001)	19.5	12.1* (0.002)
8	Delavan	8.7	8.3*(0.000)	29.8	9.7* (0.025)
9	Dickie	4.5	3.5*(0.004)	20.2	3.2(0.843)
10	Eagle	5.4	4.6*(0.001)	21.1	3(0.635)
11	Ekoln	10.0	12.3*(0.000)	27.1	10.5* (0.004)
12	Erken	10.4	11.0*(0.000)	31.8	6.8* (0.018)
13	Falling-Creek	6.9	3.0*(0.001)	27.8	-2(0.761)
14	Feeagh	8.7	5.5(0.093)	24.2	9.4(0.052)
15	Fish	5.9	5.0*(0.000)	22.5	3.9(0.421)
16	Green	4.6	5.5*(0.000)	12.6	6.8* (0.015)
17	Harp	3.6	1.6*(0.019)	15.9	-4.5(0.343)
18	Kinneret	2.9	4.8*(0.000)	7.0	9.1* (0.001)
19	Laramie	5.1	1.8(0.096)	23.7	2.7(0.762)
20	Lower-Zurich	0.9	1.0*(0.01)	2.3	1.8(0.111)
21	Mozhaysk	9.0	7.2*(0.000)	31.3	4.7(0.160)
22	Mueggelsee	16.2	16.0*(0.000)	55.3	25.2* (0.000)
23	Neuchatel	1.1	1.0*(0.005)	2.9	1.6(0.158)
24	Nohipalo-Mustjaerv	10.4	8.8*(0.000)	36.1	6.5* (0.048)
25	Nohipalo-Valgejaerv	9.3	6.6*(0.000)	32.2	-1.6(0.771)
26	Okauchee	5.3	5.3*(0.000)	18.0	6.1(0.211)
27	Paaijarvi	7.7	4.8*(0.001)	28.6	-2.5(0.927)
28	Rappbode	4.8	4.7*(0.002)	12.2	5.1(0.092)
29	Rimov	6.4	6.0*(0.001)	18.8	2.6(0.361)
30	Rotorua	7.9	11.1*(0.000)	23.1	21.8* (0.000)
31	Sparkling	4.5	4.0*(0.002)	17.8	2.4(0.618)
32	Stechlin	3.9	4.4*(0.000)	10.0	5.3* (0.027)
33	Sunapee	6.4	3.9*(0.003)	25.4	-3.2(0.563)
34	Toolik	8.1	0.7(0.662)	32.8	-7.1* (0.007)
35	Trout-Bog	4.8	1.8(0.101)	22.5	-2.2(0.060)
36	Trout-Lake	4.1	3.9*(0.001)	14.9	3.5(0.506)
37	Two-Sisters	10.2	8.2*(0.000)	35.0	8(0.145)
38	Vendyurskoe	9.8	7.6*(0.000)	37.0	6.3* (0.023)
39	Vortsjaerv	17.8	17.4*(0.000)	59.1	23.4* (0.000)
40	Washington	2.7	2.3(0.311)	7.1	3.8(0.893)
41	Windermere	5.9	4.3*(0.018)	16.2	5.3(0.099)

42	Wingra	11.7	6.8*(0.000)	53.4	6.5(0.152)
43	Mendota Lake	10.9	11.3(0.198)	33.6	13.4(0.869)
44	Bassenthwaite Lake	16.2	5.3(0.229)	56.4	2.4(0.796)
45	Esthwaite Water	10.0	-1.3(0.843)	27.2	4.7(0.508)
46	Blelham Tarn	6.7	1.6(0.351)	17.6	-0.6(0.481)
47	Selincuo (Siling) Lake	2.8	1.7(0.220)	23.1	-5.1(0.319)
48	Paikucuo Lake	9.1	-4.4(0.886)	42.3	30.1(0.063)

Supplementary Table 5. Annual mean escape depth of aquatic to escape surface heatwave events during the period of 1981-2020 and their changes between the period of 1981-2000 and 2001-2020. The "*" after the numbers indicates the temporal changes are statistically significant (*P*<0.05). The significance level of the temporal changes was tested using a one-sided t-test on the linear slope coefficient. The exact *P* values are also shown in parentheses. No adjustments were made for multiple comparisons.

Lake ID	Lake name	Escape depth (m)	Relative escape depth (%)	Δ Escape depth (m)	Δ Relative escape depth (%)
1	Allequash	3.4	57.3	0.1(0.299)	1.5(0.299)
2	Annecy	9.8	16.1	1.7(0.193)	2.8(0.193)
3	Annie	5.7	31.9	0.4(0.058)	2.1(0.058)
4	Biel	9.4	13.2	3.4(0.075)	4.8(0.075)
5	Black-Oak	8.0	31.9	1.7* (0.014)	6.9* (0.014)
6	Bourget	16.2	11.5	-1.9(0.801)	-1.4(0.801)
7	Burley-Griffin	4.2	26.4	0.2(0.326)	1.4(0.326)
8	Delavan	5.7	35.6	0.3(0.886)	1.9(0.886)
9	Dickie	3.8	35.0	0.2(0.580)	1.7(0.58)
10	Eagle	8.5	25.0	1.2(0.386)	3.5(0.386)
11	Ekoln	8.2	22.7	-0.8(0.384)	-2.3(0.384)
12	Erken	5.6	28.2	0.9(0.277)	4.3(0.277)
13	Falling-Creek	2.7	30.5	0.4* (0.001)	4.3* (0.001)
14	Feeagh	8.7	18.9	2.6(0.085)	5.6(0.085)
15	Fish	4.6	25.4	1.0* (0.000)	5.4* (0.000)
16	Green	12.5	17.6	-1.5(0.281)	-2.1(0.281)
17	Harp	5.1	13.7	1.1(0.117)	3(0.117)
18	Kinneret	10.1	24.7	0.9(0.523)	2.1(0.523)
19	Laramie	2.0	33.0	0.2* (0.011)	4.1* (0.011)
20	Lower-Zurich	12.6	9.6	3.8(0.101)	2.9(0.101)
21	Mozhaysk	4.9	24.5	0.3(0.332)	1.5(0.332)
22	Mueggelsee	2.7	34.0	0.1(0.290)	1.6(0.290)
23	Neuchatel	21.4	14.2	6.9(0.289)	4.5(0.289)
24	Nohipalo-Mustjaerv	2.2	27.2	0.2(0.060)	3(0.060)
25	Nohipalo-Valgejaerv	3.9	32.1	0.7* (0.009)	6.2* (0.009)
26	Okauchee	5.8	21.3	1.4* (0.020)	5.3* (0.02)
27	Paaijarvi	7.7	10.9	-0.6(0.939)	-0.8(0.939)
28	Rappbode	13.6	16.8	-1.8(0.385)	-2.2(0.385)
29	Rimov	6.2	14.0	2.4*(0.003)	5.5* (0.003)
30	Rotorua	7.3	14.3	3.4* (0.000)	6.6* (0.000)
31	Sparkling	6.6	36.6	0.4(0.336)	2.4(0.336)
32	Stechlin	10.3	16.8	-1.2(0.827)	-1.9(0.827)
33	Sunapee	7.4	21.9	1.6(0.096)	4.6(0.096)
34	Toolik	5.0	20.1	0.8(0.196)	3.2(0.196)
35	Trout-Bog	1.9	26.8	0.3* (0.005)	4.8* (0.005)
36	Trout-Lake	9.0	28.0	-0.2(0.444)	-0.7(0.444)
37	Two-Sisters	7.1	39.2	0.6(0.111)	3.3(0.111)
38	Vendyurskoe	3.9	30.0	0.5(0.105)	3.6(0.105)
39	Vortsjaerv	2.6	51.4	-0.1(0.434)	-1.7(0.434)
40	Washington	12.8	20.9	0.1(0.635)	0.1(0.635)
41	Windermere	8.1	19.8	0.4(0.536)	0.9(0.536)
42	Wingra	1.9	62.8	0.1(0.152)	1.9(0.152)
43	Mendota Lake	7.5	37.7	0.1(0.912)	0.3(0.912)
44	Bassenthwaite Lake	8.0	44.6	0.2(0.906)	0.9(0.906)
45	Esthwaite Water	5.5	50.0	-0.6(0.344)	-5.8(0.344)

46	Blelham Tarn	4.8	39.7	-0.4(0.486)	-3.6(0.486)
47	Selincuo (Siling) Lake	11.7	30.9	1.6(0.889)	4.3(0.889)
48	Paikucuo Lake	16.3	40.8	5.6(0.058)	13.9(0.058)

Supplementary Table 6. Annual mean number of lake bottom heatwaves without surface heatwaves during the period of 1981-2020 and their changes between the period of 1981-2000 and 2001-2020. The "*" after the numbers indicates the temporal changes are statistically significant (*P*<0.05). The significance level of the temporal changes was tested using a one-sided t-test on the linear slope coefficient. The exact *P* values are also shown in parentheses. No adjustments were made for multiple comparisons.

Lake ID	Lake name	number of lake bottom heatwaves without surface heatwaves (days)	Δ number of lake bottom heatwaves without surface heatwaves (days)	% of number of lake bottom heatwaves without surface heatwaves relative to total days of bottom heatwaves (%)	Δ % of number of lake bottom heatwaves without surface heatwaves relative to total days of bottom heatwaves (%)
1	Allequash	20.3	11.3* (0.000)	45.2	3.2* (0.023)
2	Annecy	24.6	10.0* (0.009)	25.2	8.0* (0.003)
3	Annie	30.0	-2.8(0.756)	36.5	12.1* (0.045)
4	Biel	24.5	12.4* (0.02)	31.8	12.2* (0.001)
5	Black-Oak	21.9	9.8(0.059)	39.1	1.8(0.727)
6	Bourget	26.0	16.9* (0.009)	27.5	4.0 (0.050)
7	Burley-Griffin	20.7	2.5(0.711)	52.3	-3.2(0.119)
8	Delavan	18.2	0.5(0.825)	35.9	0.9(0.921)
9	Dickie	20.7	6.3(0.051)	39.8	8.4* (0.011)
10	Eagle	22.7	5.5(0.084)	36.7	13.3* (0.001)
11	Ekoln	19.7	11.7(0.062)	35.5	1.8(0.654)
12	Erken	18.9	9.9* (0.018)	38.8	8.6* (0.031)
13	Falling-Creek	17.7	10.4* (0.003)	51.7	11.6(0.071)
14	Feeagh	21.1	4.5(0.476)	36.6	-1.2(0.512)
15	Fish	21.2	5.4(0.28)	38.6	0(0.843)
16	Green	21.6	7.6(0.085)	35.1	7.5(0.224)
17	Harp	26.9	6.5(0.467)	40.9	6.1(0.172)
18	Kinneret	22.7	21.9* (0.001)	30.1	15.7* (0.000)
19	Laramie	22.7	9.8* (0.001)	53.8	8.5* (0.011)
20	Lower-Zurich	24.3	15.2* (0.001)	22.5	10.7* (0.000)
21	Mozhaysk	25.5	-1.7(0.296)	47.9	-1.8(0.374)
22	Mueggelsee	6.5	6.6* (0.000)	20.5	9.5* (0.005)
23	Neuchatel	24.7	19.8* (0.005)	24.8	2.7(0.075)
24	Nohipalo-Mustjaerv	22.7	6.2(0.183)	43.1	5.4(0.211)
25	Nohipalo-Valgejaerv	23.4	12.4* (0.006)	43.7	14.0* (0.005)
26	Okauchee	27.7	-4.5(0.571)	41.4	-1.1(0.515)
27	Paaijarvi	33.5	-2.2(0.817)	45.9	11.0* (0.016)
28	Rappbode	29.5	4.2(0.217)	36.9	3.2(0.250)
29	Rimov	28.9	-4.1(0.856)	39.3	2.8(0.208)
30	Rotorua	15.2	9.2* (0.000)	44.7	8.9(0.077)
31	Sparkling	24.8	2.3(0.615)	40.3	12.9* (0.037)
32	Stechlin	23.8	7.6(0.077)	29.8	7.5* (0.036)
33	Sunapee	26.8	2.8(0.458)	46.4	9.7* (0.047)
34	Toolik	27.5	-3.5(0.731)	52.4	0.1(0.538)
35	Trout-Bog	23.4	6.8(0.171)	36.9	13.2* (0.011)
36	Trout-Lake	25.0	3.9(0.495)	42.3	2.9(0.217)
37	Two-Sisters	16.4	12.1* (0.001)	34.7	10.2* (0.044)
38	Vendyurskoe	21.3	0.1(0.889)	44.9	-1.0 (0.466)
39	Vortsjaerv	9.3	1.3(0.131)	29.2	-7.3(0.137)
40	Washington	25.7	22.0* (0.036)	18.3	7.9* (0.023)
41	Windermere	23.9	-3.3(0.775)	32.1	4.0(0.253)
42	Wingra	12.9	4.1* (0.002)	44.4	-2.9(0.474)

43	Mendota Lake	0.0	0.0(/)	0.0	0.0(/)
44	Bassenthwaite Lake	12.0	4.7(0.453)	37.7	7.4(0.899)
45	Esthwaite Water	29.4	-33.1(0.097)	62.4	1.8(0.640)
46	Blelham Tarn	22.0	8.7(0.128)	60.9	-25.4(0.610)
47	Selincuo (Siling) Lake	0.0	0.0(/)	0.0	0.0(/)
48	Paikucuo Lake	23.7	-1.6(0.457)	86.1	-17.4(0.070)

Supplementary Table 7. Annual mean number of concurrent lake surface and bottom heatwaves during the period of 1981-2020 and their changes between the period of 1981-2000 and 2001-2020. The "*" after the numbers indicates the temporal changes are statistically significant (*P*<0.05). The significance level of the temporal changes was tested using a one-sided t-test on the linear slope coefficient. The exact *P* values are also shown in parentheses. No adjustments were made for multiple comparisons.

Lake ID	Lake name	number of concurrent lake surface and bottom heatwaves (days)	Δ number of concurrent lake surface and bottom heatwaves (days)	% of number of concurrent lake surface and bottom heatwaves relative to total days of surface heatwaves (%)	Δ % of number of concurrent lake surface and bottom heatwaves relative to total days of surface heatwaves (%)
1	Allequash	7.5	7.2*(0.000)	27.2	15.6*(0.001)
2	Annecy	4.7	5.1*(0.001)	11.7	6.4*(0.004)
3	Annie	4.1	0.2(0.318)	15.1	-4.7(0.875)
4	Biel	6.4	5.8*(0.001)	17.8	8.4*(0.025)
5	Black-Oak	6.0	8.8*(0.000)	17.6	19.0*(0.000)
6	Bourget	4.0	4.6*(0.004)	10.2	7.1*(0.009)
7	Burley-Griffin	7.4	7.8*(0.000)	27.8	15.9*(0.001)
8	Delavan	7.5	7.6*(0.000)	24.7	11.5*(0.005)
9	Dickie	3.3	4.2*(0.000)	13.1	10.2*(0.004)
10	Eagle	3.4	5.0*(0.000)	10.9	12.8*(0.000)
11	Ekoln	8.8	11.6*(0.000)	22.6	12.8*(0.001)
12	Erken	8.9	11.1*(0.000)	24.0	12.8*(0.002)
13	Falling-Creek	9.1	5.0*(0.000)	35.9	1.4(0.566)
14	Feeagh	11.1	7.2(0.072)	31.3	13.7*(0.032)
15	Fish	5.5	5.4*(0.000)	20.4	8.7(0.054)
16	Green	4.2	6.1*(0.000)	11.2	10.8*(0.000)
17	Harp	2.4	3.1*(0.007)	9.0	5.8(0.121)
18	Kinneret	5.6	9.6*(0.000)	12.8	18.4*(0.000)
19	Laramie	5.6	4.4*(0.001)	24.8	13.2*(0.007)
20	Lower-Zurich	3.4	4.1*(0.000)	9.2	6.3*(0.004)
21	Mozhaysk	7.3	6.9*(0.000)	23.6	8.6*(0.007)
22	Mueggelsee	18.3	18.1*(0.000)	62.7	28.8*(0.000)
23	Neuchatel	4.2	5.7*(0.001)	9.7	9.6*(0.002)
24	Nohipalo-Mustjaerv	8.9	10.0*(0.000)	28.1	15.4*(0.000)
25	Nohipalo-Valgejaerv	6.4	8.7*(0.000)	17.4	15.3*(0.000)
26	Okauchee	5.2	5.7*(0.000)	16.1	8.5*(0.003)
27	Paaijarvi	4.1	4.2*(0.001)	12.9	3.8(0.252)
28	Rappbode	6.8	8.0*(0.000)	16.7	8.5*(0.008)
29	Rimov	5.4	6.1*(0.000)	14.2	7.9*(0.004)
30	Rotorua	9.9	12.2*(0.000)	30.1	22.0*(0.000)
31	Sparkling	3.5	4.8*(0.000)	11.0	10.1*(0.004)
32	Stechlin	4.7	7.7*(0.000)	10.5	15.9*(0.000)
33	Sunapee	5.4	5.2*(0.001)	19.6	5.9(0.214)
34	Toolik	3.3	1.7*(0.02)	12.0	2.1(0.425)
35	Trout-Bog	2.7	3.2*(0.000)	10.1	8.7*(0.000)
36	Trout-Lake	3.6	4.6*(0.005)	10.9	8.7*(0.036)
37	Two-Sisters	8.3	9.5*(0.000)	24.5	18.3*(0.000)
38	Vendyurskoe	8.2	7.2*(0.000)	29.2	8.1*(0.002)
39	Vortsjaerv	17.2	18.7*(0.000)	54.3	30.7*(0.000)
40	Washington	6.5	7.3*(0.033)	13.6	10.8(0.065)
41	Windermere	8.7	7.7*(0.002)	23.4	10.7*(0.003)

42	Wingra	12.6	7.5*(0.000)	57.1	8.3(0.118)
43	Mendota Lake	0.0	0.0(/)	0.0	0.0(/)
44	Bassenthwaite Lake	17.2	3.1(0.567)	61.7	-3.1(0.724)
45	Esthwaite Water	11.2	-4.1(0.634)	27.1	-8.0(0.551)
46	Blelham Tarn	7.1	3.9(0.187)	17.5	6.1(0.193)
47	Selincuo (Siling) Lake	0.0	0.0(/)	0.0	0.0(/)
48	Paikucuo Lake	2.8	3.3*(0.026)	13.2	28.7*(0.009)

Supplementary Table 8. Optimal parameter settings and accuracy performance of lake-specific calibrated FLake models over 6 lakes. Calibrated parameters include snow accumulation rate (Snow_rate, kg/(m^2 s)), scale and offset of lake depth (m), scale and offset of wind speed (U, m/s), albedo for snow and white ice (albedo_1), albedo for melting snow and blue ice (albedo_2), light attenuation coefficient (Kd, m^{-1}), scale of solar radiation (SR_scale), and scale of surface air temperature (SAT_scale). Accuracy performances are evaluated based on median absolute error (MAE, °C) at different depths (full depth, surface, bottom, 1/3 of total depth, and 2/3 of total depth) and different seasons (Spring, Summer, Autumn, and Winter).

Lake ID		43	44	45	46	47	48
Lake name		Mendota	Bassenthwaite	Esthwaite Water	Blelham Tarn	Selincuo (Siling)	Paikucuo
Settings	Snow_rate	0.000001	0.1	0.5	0.5	0.00001	0.0002
	Depth scale	0	0	0	1	0.75	0.5
	Depth offset	18	9	10	4	0	0
	U scale	1.25	1.5	1	1	1	1.5
	U offset	6	0	0	0	2	1
	albedo_1	0.6	0.6	0.6	0.6	0.6	0.6
	albedo_2	0.1	0.1	0.1	0.1	0.1	0.1
	Kd	0.2	0.33	0.4	0.5	0.2262	0.18
	SR_scale	1.24	1.2	1.1	1.1	1.02	1
	SAT_scale	1.009	1	1	1	1	1
MAE (℃)	Full depth	1.0	0.9	0.7	1.3	0.6	1.1
	Surface	0.8	0.8	0.6	1.0	0.7	0.9
	1/3 depth	0.8	0.9	0.5	1.5	0.5	1.2
	2/3 depth	1.3	1.0	0.9	1.4	0.7	1.1
	Bottom	1.4	1.1	1.0	1.3	0.8	1.0
	Spring	0.5	1.7	1.6	1.8	/	1.0
	Summer	1.3	0.7	1.0	1.3	1.3	1.4
	Autumn	1.1	1.0	0.5	1.1	0.6	0.7
	Winter	0.7	0.5	0.4	0.7	0.6	1.0
