# Supplementary material for: Subsurface heatwaves in lakes
Source: Nat Clim Chang. 2025 Apr 10;15(5):554–9. doi: 10.1038/s41558-025-02314-0 (PMC12064439; doi:10.1038/s41558-025-02314-0)
Supplement: Supplementary file 2 — Reporting Summary [file 41558_2025_2314_MOESM2_ESM.pdf]

nature portfolio

Corresponding author(s): RI Woolway

Last updated by author(s): Mar 4, 2025

Reporting Summary

Nature Portfolio wishes to improve the reproducibility of the work that we publish. This form provides structure for consistency and transparency in reporting. For further information on Nature Portfolio policies, see our Editorial Policies and the Editorial Policy Checklist.

Statistics

For all statistical analyses, confirm that the following items are present in the figure legend, table legend, main text, or Methods section.

n/a	Confirmed	
☐	☒	The exact sample size (n) for each experimental group/condition, given as a discrete number and unit of measurement
☒	☐	A statement on whether measurements were taken from distinct samples or whether the same sample was measured repeatedly
☒	☐	The statistical test(s) used AND whether they are one- or two-sided<br>*Only common tests should be described solely by name; describe more complex techniques in the Methods section.*
☒	☐	A description of all covariates tested
☒	☐	A description of any assumptions or corrections, such as tests of normality and adjustment for multiple comparisons
☐	☒	A full description of the statistical parameters including central tendency (e.g. means) or other basic estimates (e.g. regression coefficient) AND variation (e.g. standard deviation) or associated estimates of uncertainty (e.g. confidence intervals)
☒	☐	For null hypothesis testing, the test statistic (e.g. F, t, r) with confidence intervals, effect sizes, degrees of freedom and P value noted<br>*Give P values as exact values whenever suitable.*
☒	☐	For Bayesian analysis, information on the choice of priors and Markov chain Monte Carlo settings
☒	☐	For hierarchical and complex designs, identification of the appropriate level for tests and full reporting of outcomes
☒	☐	Estimates of effect sizes (e.g. Cohen's d, Pearson's r), indicating how they were calculated

Our web collection on statistics for biologists contains articles on many of the points above.

Software and code

Policy information about availability of computer code

Data collection	We used ISIMIP Local lake simulations (https://data.isimip.org/10.48364/ISIMIP.563533) and ISIMIP Global lake simulations (https://data.isimip.org/10.48364/ISIMIP.931371) for investigating historic and future lake conditions. We used the Freshwater Lake model (FLake) for simulating some lake temperature profiles. The source code of FLake is available here: http://www.flake.igb-berlin.de/.
Data analysis	We used R version 3.5.2. No custom algorithms or software were used.

For manuscripts utilizing custom algorithms or software that are central to the research but not yet described in published literature, software must be made available to editors and reviewers. We strongly encourage code deposition in a community repository (e.g. GitHub). See the Nature Portfolio guidelines for submitting code & software for further information.

Data

Policy information about availability of data

All manuscripts must include a data availability statement. This statement should provide the following information, where applicable:

- Accession codes, unique identifiers, or web links for publicly available datasets
- A description of any restrictions on data availability
- For clinical datasets or third party data, please ensure that the statement adheres to our policy

ISIMIP Local lake simulations: https://data.isimip.org/10.48364/ISIMIP.563533; ISIMIP Global lake simulations: https://data.isimip.org/10.48364/ISIMIP.931371; simulations of lake temperature profiles over individual lakes generated in this study are available by ref. 53. The mooring observations for the Laurentian Great

Lakes used in the model evaluation are available from https://www.glerl.noaa.gov/data/#watertemp. The heatwave metrics calculated for the Great Lakes ae available from ref. 48. Global heatwave metrics calculated from the ISIMIP projections re available from ref. 56.

Research involving human participants, their data, or biological material

Policy information about studies with human participants or human data. See also policy information about sex, gender (identity/presentation), and sexual orientation and race, ethnicity and racism.

Reporting on sex and gender	N/A
Reporting on race, ethnicity, or other socially relevant groupings	N/A
Population characteristics	N/A
Recruitment	N/A
Ethics oversight	N/A

Note that full information on the approval of the study protocol must also be provided in the manuscript.

Field-specific reporting

Please select the one below that is the best fit for your research. If you are not sure, read the appropriate sections before making your selection.

☐ Life sciences ☐ Behavioural & social sciences ☒ Ecological, evolutionary & environmental sciences

For a reference copy of the document with all sections, see nature.com/documents/nr-reporting-summary-flat.pdf

Ecological, evolutionary & environmental sciences study design

All studies must disclose on these points even when the disclosure is negative.

Study description	We calculated the characteristics of subsurface heatwaves in lakes worldwide.
Research sample	We calculated the heatwave metrics for 16,455 lakes worldwide.
Sampling strategy	The study sites are selected based on the availability of model derived lake temperature projections. The forcing data and lake models used are commonly used in lake studies.
Data collection	We modelled lake temperature profiles in this study.
Timing and spatial scale	1980-2099 at daily resolution
Data exclusions	No data were excluded.
Reproducibility	The forcing data, lake model, and modelled lake temperature profiles are publcly available.
Randomization	Not relevant as all simulation results were used in the analysis.
Blinding	Not relevant as all simulation results were used in the analysis.

Did the study involve field work? ☐ Yes ☒ No

Reporting for specific materials, systems and methods

We require information from authors about some types of materials, experimental systems and methods used in many studies. Here, indicate whether each material, system or method listed is relevant to your study. If you are not sure if a list item applies to your research, read the appropriate section before selecting a response.

Materials & experimental systems

n/a	Involved in the study
☒	☐ Antibodies
☒	☐ Eukaryotic cell lines
☒	☐ Palaeontology and archaeology
☒	☐ Animals and other organisms
☒	☐ Clinical data
☒	☐ Dual use research of concern
☒	☐ Plants

Methods

n/a	Involved in the study
☒	☐ ChIP-seq
☒	☐ Flow cytometry
☒	☐ MRI-based neuroimaging

Plants

Seed stocks

N/A

Novel plant genotypes

N/A

Authentication

N/A
